# Al(OR^F^)_3_ (R^F^ = C(CF_3_)_3_) activated silica: a well-defined weakly coordinating surface anion†

**DOI:** 10.1039/c9sc05904k

**Published:** 2019-12-19

**Authors:** Damien B. Culver, Amrit Venkatesh, Winn Huynh, Aaron J. Rossini, Matthew P. Conley

**Affiliations:** Department of Chemistry, University of California Riverside California 92521 USA matthew.conley@ucr.edu; Department of Chemistry, Iowa State University Ames Iowa 50011 USA

## Abstract

Weakly Coordinating Anions (WCAs) containing electron deficient delocalized anionic fragments that are reasonably inert allow for the isolation of strong electrophiles. Perfluorinated borates, perfluorinated aluminum alkoxides, and halogenated carborane anions are a few families of WCAs that are commonly used in synthesis. Application of similar design strategies to oxide surfaces is challenging. This paper describes the reaction of Al(OR^F^)_3_*PhF (R^F^ = C(CF_3_)_3_) with silica partially dehydroxylated at 700 °C (SiO_2-700_) to form the bridging silanol ≡Si–OH⋯Al(OR^F^)_3_ (**1**). DFT calculations using small clusters to model **1** show that the gas phase acidity (GPA) of the bridging silanol is 43.2 kcal mol^−1^ lower than the GPA of H_2_SO_4_, but higher than the strongest carborane acids, suggesting that deprotonated **1** would be a WCA. Reactions of **1** with NOct_3_ show that **1** forms weaker ion-pairs than classical WCAs, but stronger ion-pairs than carborane or borate anions. Though **1** forms stronger ion-pairs than these state-of-the-art WCAs, **1** reacts with alkylsilanes to form silylium type surface species. To the best of our knowledge, this is the first example of a silylium supported on derivatized silica.

## Abstract 2

A strong Lewis acid coordinates to silanols on silica to form a strong acid, which acts as a weakly coordinating anion when deprotonated.

## Graphical abstract

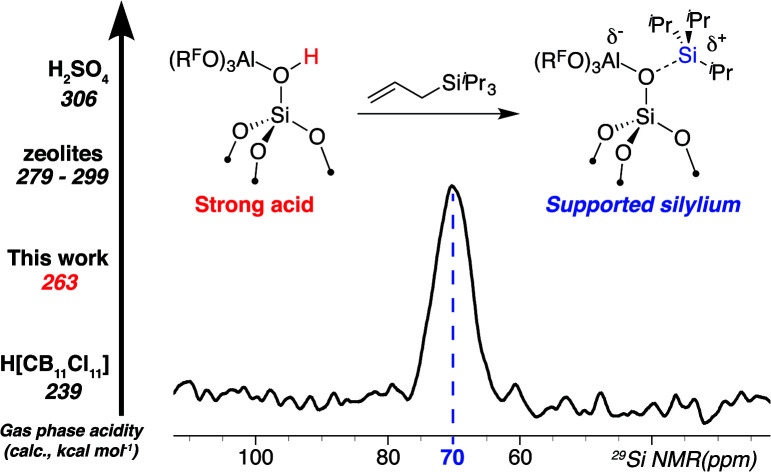

## Introduction

**Fig. 1:**
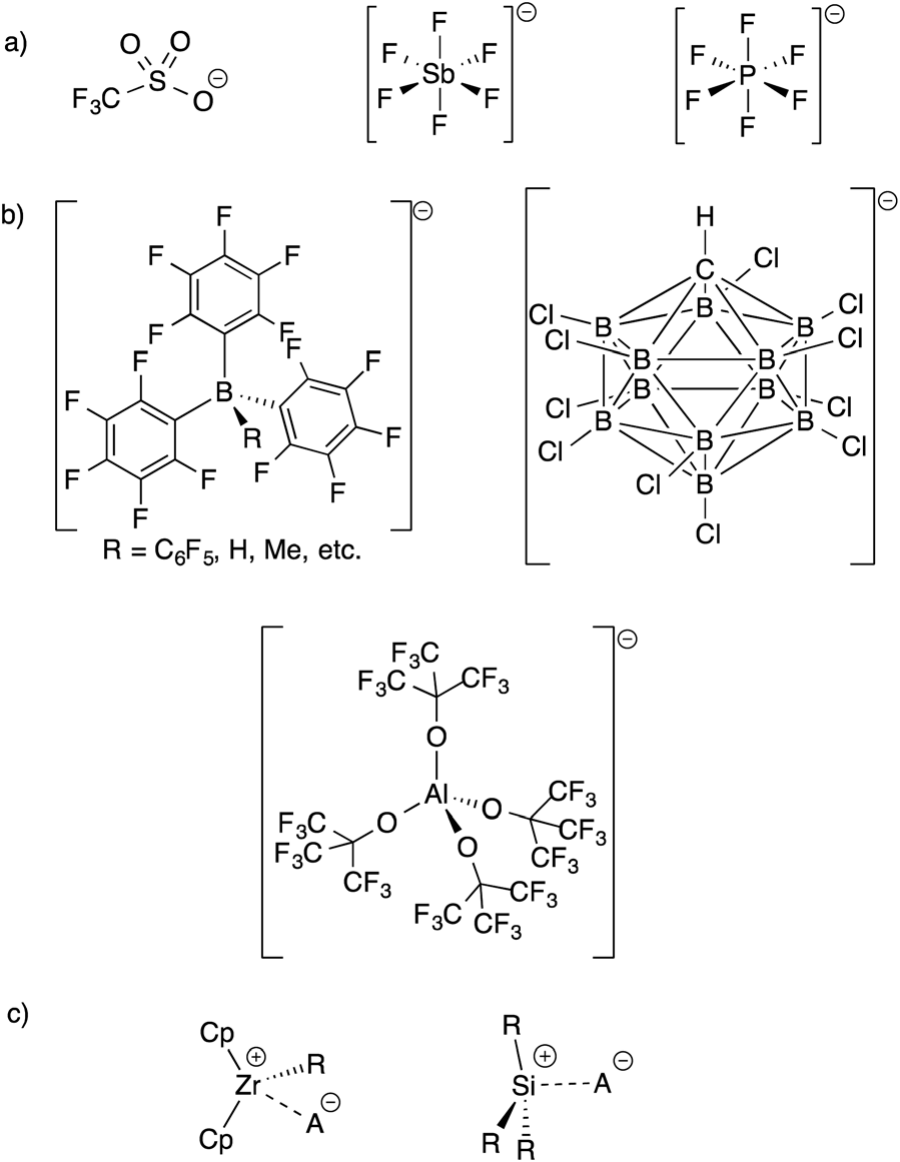
Structures of first generation WCAs (a) and bulky inert WCAs (b); very strong electrophiles that are too reactive to form with first generation WCAs (c).

The development of inert Weakly Coordinating Anions (WCAs) was critical to isolate very reactive electrophilic species.^1^ Studies of superacid media resulted in the first generation of WCAs (CF_3_SO_3_^−^, PF_6_^−^, SbF_6_^−^, *etc.*, Fig. 1).^2^ The first generation WCAs continue to find broad applications in the synthetic community, but these anions are too reactive or coordinating to stabilize highly reactive cations. For example, organometallic Zr(iv) cations, key 14-electron intermediates in the synthesis of polyolefins, are incompatible with first generation WCAs.^3^ These anions are also not sufficiently weakly coordinating to form R_3_Si^+^ cations.^4^

Fluorinated borates (*e.g.*^−^B(C_6_F_5_)_4_, ^−^B(3,5-(CF_3_)_2_–C_6_H_3_)_4_),^5^ aluminates (*e.g.*^−^Al(OC(CF_3_)_3_)_4_),^6^ or carborane anions (*e.g.*^−^CHB_11_H_6_X_5_, ^−^CHB_11_X_11_; X = halide),^7^ shown in Fig. 1, stabilize organometallic Zr(iv) cations or R_3_Si^+^. The anions are designed to delocalize charge throughout the structure of the WCA, which results in low basicity. The conjugate acids of the WCAs shown in Fig. 1, when isolable, are the strongest known Brønsted acids.^8^ The strong C–F or B–X bonds in these WCAs also provide some degree of chemical inertness, which is important in reactions involving the strong electrophiles mentioned above.

**Fig. 2:**
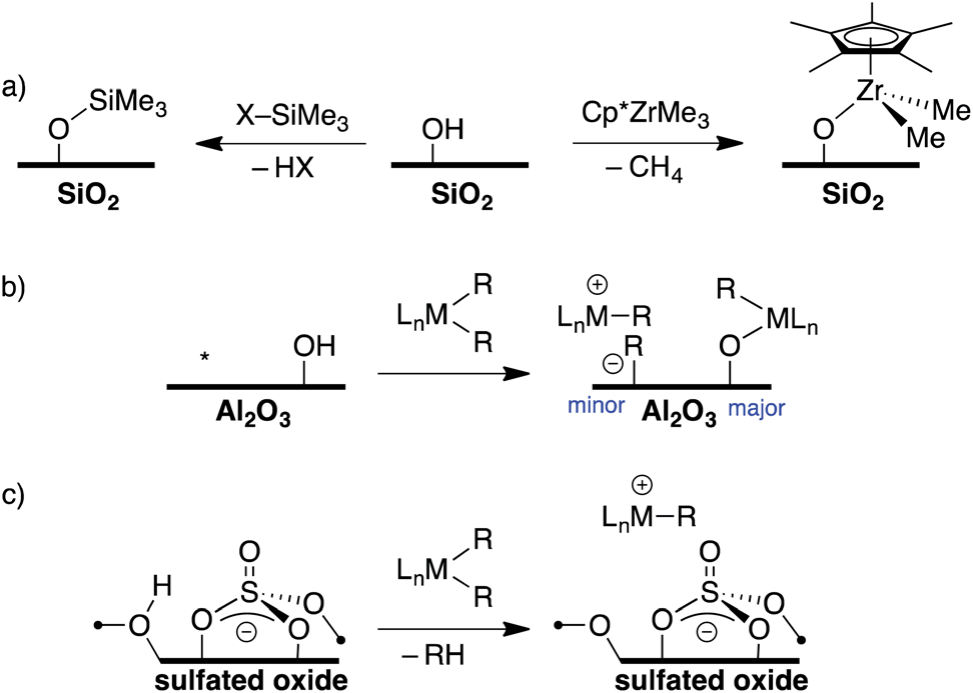
Formation of R_3_Si–O_*x*_ and Cp*Zr(Me)_2_–O_*x*_ (O_*x*_ = surface oxygen) on partially dehydroxylated SiO_2_ (a); reaction of organometallics with partially dehydroxylated Al_2_O_3_ (* = Lewis acid site), minor product are ion-pairs (b); formation of electrophilic ion pairs on sulfated oxides (c).

Direct translation of these concepts to well-defined heterogeneous catalysts is more challenging. Well-defined heterogeneous catalysts are desirable because the molecular structure of a catalytically active site can be determined using spectroscopic methods,^9–11^ which provides opportunities to optimize the properties of these catalysts based on the structure of the active site. The largest class of well-defined heterogeneous catalysts are supported on SiO_2_ partially dehydroxylated at 700 °C. Well-defined sites supported on SiO_2_ generally do not form ion-pairs but rather ≡SiO–ML_*n*_. For example, the ^29^Si Cross Polarization Magic Angle Spinning (CPMAS) NMR spectrum of alkylsilane functionalized silica (≡SiO–SiMe_3_, Fig. 2a) contains a signal at 14 ppm for the alkylsilane fragment, which is inconsistent with formation of a Me_3_Si^+^ species on the silica surface.^12–15^ Similarly, Cp*ZrMe_3_ (Cp* = pentamethylcyclopentadienyl) reacts with partially dehydroxylated SiO_2_ to form ≡SiO–Zr(Cp*)Me_2_ (Fig. 2a),^16^ which is inactive in the polymerization of ethylene. However, ≡SiO–Zr(Cp*)Me_2_ does react with B(C_6_F_5_)_3_ to form electrophilic ion-pairs that are active in the polymerization of ethylene.^17^ Silica surfaces can also form strong ion-pars with between surface siloxide anions and tetraalkylphosphonium groups.^18,19^

Partially dehydroxylated Al_2_O_3_ contains a very small quantity of tri-coordinate Al Lewis-acid sites^20^ that react with organometallic complexes to form electrophilic ion-pairs, Fig. 2b.^21,22^ However, the surface coverage of the –OH sites is much higher than the surface coverage of Lewis sites, resulting in low active site loadings in these well-defined catalysts.^23^

The trends in WCAs described above suggest that oxides containing more acidic –OH sites may be more weakly coordinating. Zeolites contain –OH sites that are more acidic than –OH sites on SiO_2_, and can support organometallic species.^24^ Studies of well-defined organometallics are limited to small molecules because SiO_2_/Al_2_O_3_ zeolite materials have small pore sizes. Oxides treated with sulfuric acid, sulfated oxides, were claimed to contain superacid Brønsted acid sites.^25^ This relates sulfated oxides to first generation WCAs, and several studies showed that sulfated oxides form electrophilic ion pairs with organometallics (Fig. 2c).^26–31^ However, titrations of the –OH sites on sulfated oxides with phosphines are inconsistent with superacid –OH sites.^32^ This data is consistent with DFT calculations showing that sulfated oxides are weaker acids than zeolites.^33^ Detailed studies showed that sulfated oxides also contain significant amounts of oxidative pyrosulfate sites, which can result in undesirable side reactions with organometallic substrates.^34^

**Fig. 3:**
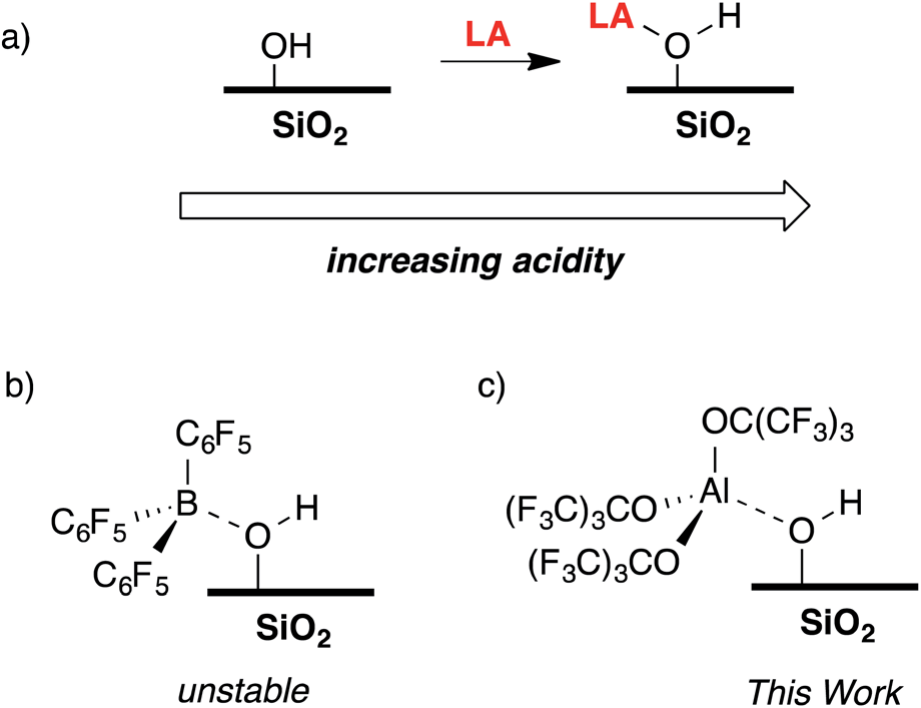
The reaction of a Lewis acid with silica to form a bridging silanol (a); B(C_6_F_5_)_3_ reacts with silica to form unstable bridging silanols (b); the focus of this work, generation of ≡Si–OH⋯Al(OR^F^)_3_ (c).

The reaction of a strong Lewis acid and a ≡Si–OH on partially dehydroxylated silica should form a strong Brønsted acid site (Fig. 3a).^35^ Deprotonation of the strong Brønsted acid should result in a weakly coordinating anion that may stabilize electrophilic surface species that would not typically form on SiO_2_ surfaces, and also translate solution WCA concepts to heterogeneous supports. Contacting dehydroxylated silica with AlCl_3_ forms strong Brønsted acid sites, but also results in various side reactions leading to strong Lewis sites on the silica surface,^36^ which is common in this class of functionalized oxides.^37^ Redox inactive strong Lewis acids, such as B(C_6_F_5_)_3_, are not sufficiently Lewis acidic to form stable bridging silanols with silica (Fig. 3b).^38^ However, B(C_6_F_5_)_3_ reacts with silica and aniline bases to form ion-pairs that are capable of activating organometallic species,^39,40^ or with exogenous H_2_O to form grafted species on the SiO_2_ surface.^41^ This paper describes the reaction of Al(OR^F^)_3_*PhF (R = C(CF_3_)_3_)^42^ with silica partially dehydroxylated at 700 °C (SiO_2-700_) to generate ≡Si–OH⋯Al(OR^F^)_3_ (**1**, Fig. 3c). Calculated gas phase acidity (GPA) of **1** shows that the activated silanols are very strong Brønsted acids. Reactions of **1** with silane reagents result in the formation of [R_3_Si][≡Si–O⋯Al(OR^F^)_3_], a rare example of a silylium supported on SiO_2_.

## Results and discussion

### Reaction of Al(OR^F^)_3_*PhF with partially dehydroxylated SiO_2_

**Fig. 4:**
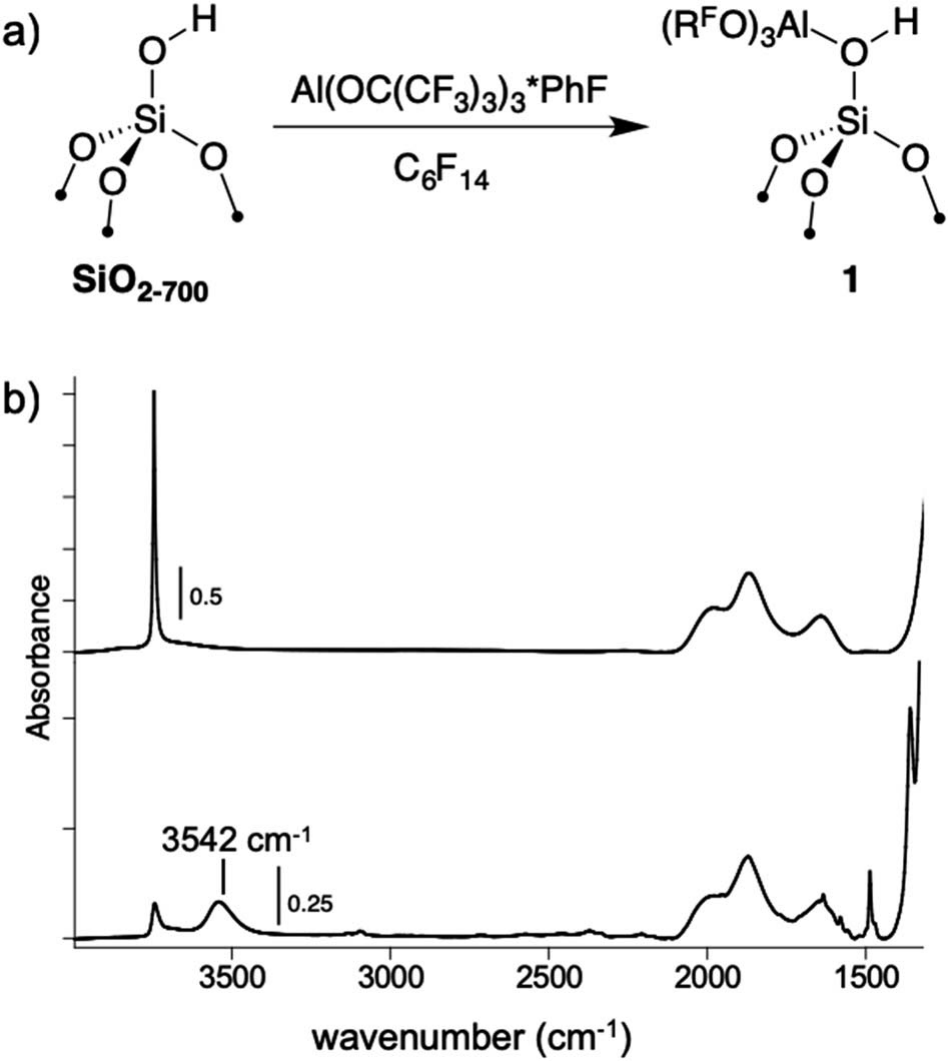
The reaction of Al(OR^F^)_3_*PhF with SiO_2-700_ in perfluorohexane (a); FTIR spectra of SiO_2-700_ (top) and **1** (bottom, b).

A perfluorohexane slurry of SiO_2-700_ (0.26 mmol OH g^−1^) reacts with Al(OR^F^)_3_*PhF to form ≡Si–OH⋯Al(OR^F^)_3_ (**1**, Fig. 4a). ICP-OES analysis shows that 0.24 mmol g^−1^ is present in **1**, indicating that most of the silanols in SiO_2-700_ are coordinated to Al(OR^F^)_3_. The FTIR spectrum of **1**, shown in Fig. 4b, contains a new red-shifted *ν*_OH_ at 3542 cm^−1^ that is typical of bridging silanols in silica–alumina materials. This spectrum also contains a *ν*_OH_ corresponding to silanols that do not form adducts with Al(OR^F^)_3_. Weak sp^2^–*ν*_CH_ and *ν*_C=C_ are also present, suggesting that some fluorobenzene remains adsorbed to **1**. Consistent with this observation, ^19^F{^1^H} NMR measurements of **1** suspended in CD_3_CN show that 0.045 ± 0.004 mmol g^−1^ of PhF leaches off the silica surface (Fig. S19†).

**Fig. 5:**
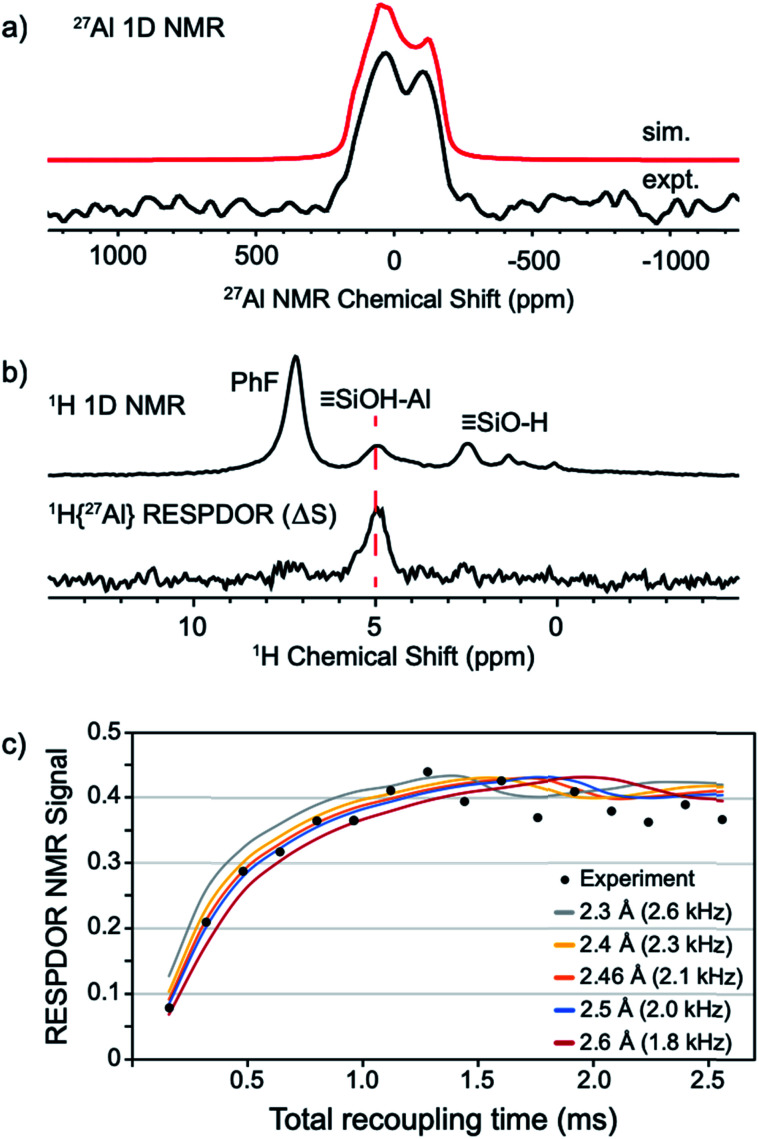
Static ^27^Al solid-state NMR spectrum of **1** acquired at 14.1 T (a); solid-state 1D ^1^H MAS NMR (top) and ^1^H{^27^Al} RESPDOR difference spectrum of **1** (middle, b); fit of RESPDOR dipolar dephasing curve to measure dipolar ^1^H–^27^Al dipolar coupling present in **1** (bottom, c). See the ESI† for Experimental details.

The static ^27^Al NMR spectrum of **1** contains a typical quadrupolar powder pattern that can be simulated with a single site (Fig. 5a).^43^ The isotropic chemical shift (*δ*_iso_ = 43 ppm) and large quadrupolar coupling constant (*C*_Q_ = 14.6 MHz) is consistent with a highly distorted tetrahedral Al coordination environment. These values are in agreement with those obtained from ^27^Al MAS measurements of **1** (Fig. S4†). The ^1^H magic angle spinning (MAS) NMR spectrum of **1** contains signals at 7.1 (adsorbed PhF), 5.0 (≡Si–OH⋯Al(OR^F^)_3_), and 2.3 (≡Si–OH) ppm (Fig. 5b, top trace). A ^1^H dipolar double-quantum single-quantum (DQ-SQ) spectrum does not show crosspeaks between adsorbed PhF and the bridging silanol (see the ESI, Fig. S5†), suggesting that adsorbed PhF is distant from the acidic silanol in **1**. A 2D ^1^H{^27^Al} D-RINEPT spectrum shows that the ^27^Al signal in **1** correlates to the acidic silanol (≡Si–OH⋯Al(OR^F^)_3_) signal at 5.0 ppm (Fig. S7†), supporting these assignments.

The ^1^H{^27^Al} Resonance-Echo Saturation-Pulse Double-Resonance (RESPDOR)^44,45^ NMR experiment allows measurement of the dipolar coupling constant for ^1^H and ^27^Al spins. The ^1^H–^27^Al dipolar coupling constant is inversely proportional to the cube of the inter-atomic distance, so only ^1^H and ^27^Al spins that are in close spatial proximity (<5 Å) will be affected in this experiment. The ^1^H{^27^Al}-RESPDOR difference NMR spectrum (Δ*S*) is shown in Fig. 5b (bottom) and contains a single ^1^H NMR signal at 5.0 ppm (≡Si–OH⋯Al(OR^F^)_3_). This result indicates that the bridging silanol is close to the aluminum in Al(OR^F^)_3_, and that the signals at 7.1 ppm (PhF) and 2.3 ppm (≡Si–OH) are from protons distant from aluminum, as expected. Variation of recoupling times in the ^1^H{^27^Al}-RESPDOR pulse sequence, and numerical simulation of the RESPDOR dipolar dephasing curve allows the ^1^H–^27^Al dipolar coupling constant to be determined. These data are given in Fig. 5c, and show that the ^1^H–^27^Al dipolar coupling is ∼2.0–2.3 kHz, which corresponds to Al–OH distances in the range of 2.4–2.5 Å. This distance is in good agreement with structural models predicted by DFT (see below).

### DFT studies of small cluster models of **1**

**Fig. 6:**
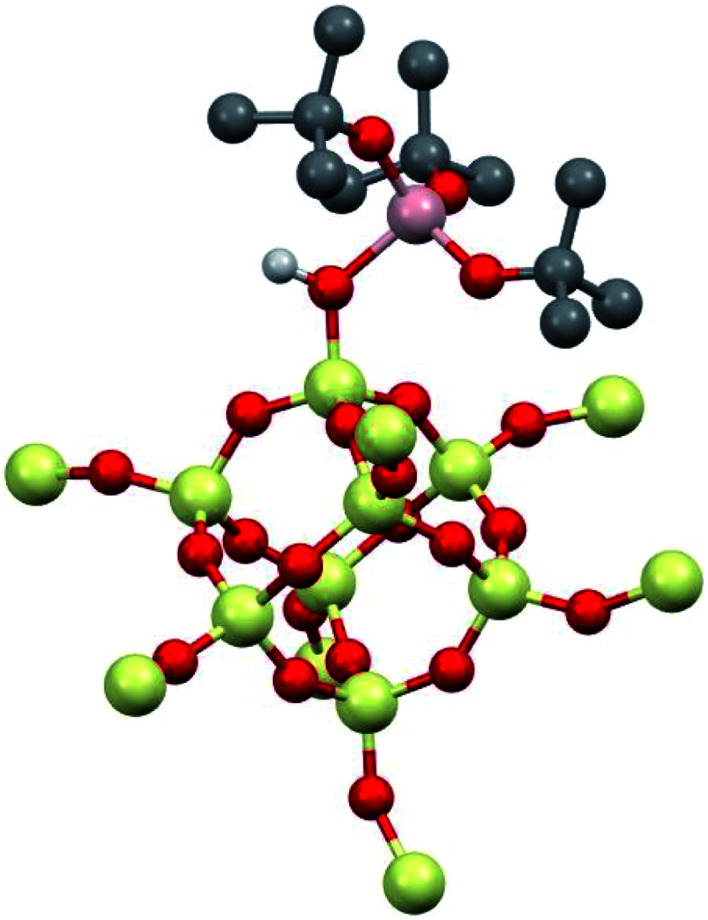
**1-DFT** with selected hydrogens and fluorines hidden for clarity. Selected distances (Å) and angles (deg.): O–H (0.98 Å), Al–OH (1.91 Å), Al–OH (2.46 Å) Al–OR^F^ (1.74 Å), Si–OH (1.70 Å), Si–O_cluster_ (1.60–1.63 Å), H–O–Al 113°, H–O–Si 116°, Si–O–Al 128°, O–Si–O(H) 105°.

**1** was modeled using Al(OR^F^)_3_ and the –SiH_3_ capped polysesquisiloxane cluster^46^ at the B3LYP/6-31G(d,p) level of theory. The cluster **1-DFT** is shown in Fig. 6. Al(OR^F^)_3_ in **1-DFT** coordinates to the isolated silanol in the cluster and not Si–O–Si bridges. The aluminum fragment in **1-DFT** adopts a distorted tetrahedral geometry, and the Al–OH distance in **1-DFT** is 1.91 Å. The terminal Si–O distance is 1.70 Å, slightly longer than the average Si–O distances (1.62 Å) in the cluster. These observations are similar to those obtained for alcohol adducts of Al(OR^F^)_3_.^47^ The predicted Al–OH distance is 2.46 Å, and is in good agreement with an estimated Al–OH distances determined with the ^1^H{^27^Al} RESPDOR experiment.

The calculated IR spectrum of **1-DFT** predicts a *ν*_OH_ at 3550 cm^−1^ (expt. ν_OH_ = 3542 cm^−1^). NMR calculations at the M06L/Al(6-311G(d,p)), 6-31G(d,p) level of theory predict that the acidic proton appears at 5.1 ppm, and that the ^27^Al *C*_Q_ is 15.3 MHz. These values agree well with those obtained experimentally for **1**, and are similar to those obtained for molecular H[Al(OC(CF_3_)_3_)_4_].^47^

Quantitative measurement of Brønsted acidity on oxides is challenging.^48–50^ Gas-phase acidity (GPA) can be calculated using DFT methods, and is reasonably accurate for small molecules. Table 1 gives the GPA of various mineral acids at BP86/def2-TZVP to calibrate the accuracy of this level of theory. The GPA of HCl is 334.5 kcal mol^−1^, which is very close to the experimental value (333.6 kcal mol^−1^). In general, we find good agreement between experimental and calculated values. The calculated deprotonation energy of **1-DFT** is 262.7 kcal mol^−1^. For comparison, small clusters of ≡Si–OH⋯Al(OMe)_3_, simplified models for bridging silanols in SiO_2_/Al_2_O_3_, were also calculated at this level of theory and have deprotonation energy of 279–299 kcal mol^−1^ (see the ESI† for details). These values are similar to those calculated for more complex models of zeolities,^51–54^ indicating that **1-DFT** is more acidic than bridging silanols in silica/aluminas. However, **1-DFT** is clearly a weaker acid than H[Al(OC(CF_3_)_3_)_4_] (GPA = 262.7 kcal mol^−1^) or the H[CHB_11_Cl_11_] carborane acid (GPA = 239.0 kcal mol^−1^). The strong Brønsted acidity of **1** suggests that the conjugate base of the bridging silanol may behave as a weakly coordinating anion.

**Table 1:** Calculated gas-phase acidity (GPA) in kcal mol^−1^ at BP86/def2-TZVP level of theory

Acid	Expt. GPA (kcal mol^−1^)	Calc'd GPA (kcal mol^−1^)
HCl	336.2	334.5
HBr	318.3	321.6
HI	309.2	305.9
H_2_SO_4_	302.2	305.9
HSO_3_F	299.8	294.6
Zeolite	—	279–299
HSO_3_CF_3_	299.5	293.3
**1-DFT**	—	262.7
H[Al(OC(CF_3_)_3_)_4_]	—	248.8 (ref. 47)
H[CHB_11_Cl_11_]	—	239.1

### Formation of ion-pairs with **1**

The most common experimental method to assess the ion-pairing on a solid involves adsorption of a probe molecule to the solid and measuring the change in a spectroscopic observable, usually Δ*ν* by FTIR or Δ*δ* by NMR spectroscopy. However, solution ^19^F{^1^H} NMR studies indicate adsorption of common probes (pyridine or triethylphosphine oxide) or heteroatom containing solvents (CD_3_CN, Et_2_O, or CH_2_Cl_2_) to **1** results in desorption of solvated Al(OR^F^)_3_ from the silica surface.

Reed and co-workers described the properties of [Oct_3_NH][X] contact ion pairs in CCl_4_ solution.^55^ The *ν*_NH_ stretch from FTIR measurements provides information about ion-pairing in [Oct_3_NH][X]. In a H-bonded contact ion-pair, weaker NH⋯X interactions will result in higher *ν*_NH_ stretching frequencies. The *ν*_NH_ values for selected [Oct_3_NH][X] contact ion pairs in CCl_4_ solution are given in Table 2.

**Table 2:** *ν*_NH_ stretching frequencies for [Oct_3_NH][X]

Anion	*ν*_NH_ a (cm^−1^)
B(C_6_F_5_)_4_	3223
[CHB_11_Cl_11_]	3163
[CH_5_B_11_Cl_6_]	3148
[CH_5_B_11_Br_6_]	3125
[CH_5_B_11_I_6_]	3097
**2**	3070^b^
ClO_4_	3049
FSO_3_	2953
CF_3_SO_3_	2939

a Values from ref. 54.

b This work.

The reaction of **1** with 0.95 equiv NOct_3_ in C_6_H_12_ at room temperature results in the formation of [Oct_3_NH][(R^F^O)_3_Al–OSi≡] (**2**, eqn (1)). Solution ^19^F NMR spectra of this mixture indicates that desorption of Al(OR^F^)_3_ or decomposition of **1** does not occur under these conditions. **2** was characterized by multinuclear solid-state NMR spectroscopy (see the ESI† for details).1
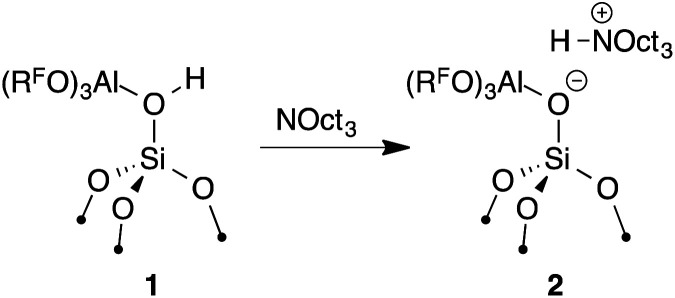


The FTIR spectrum of **2** shows *ν*_NH_ at 3070 cm^−1^. This result indicates **2** forms a weaker ion pair than [Oct_3_NH][SO_3_CF_3_] (*ν*_NH_ = 2939 cm^−1^) or [Oct_3_NH][ClO_4_] (*ν*_NH_ = 3049 cm^−1^), common first generation WCAs. This experimental data is consistent with the calculated GPA showing that **2** is a very strong Brønsted acid because strong acids form weak ion pairs. However, **2** forms stronger ion-pairs with [Oct_3_NH] than carborane or [B(C_6_F_5_)_4_] anions.

### Formation of [^i^Pr_3_Si][(R^F^O)_3_Al–OSi≡] (**3**)

**Fig. 7:**
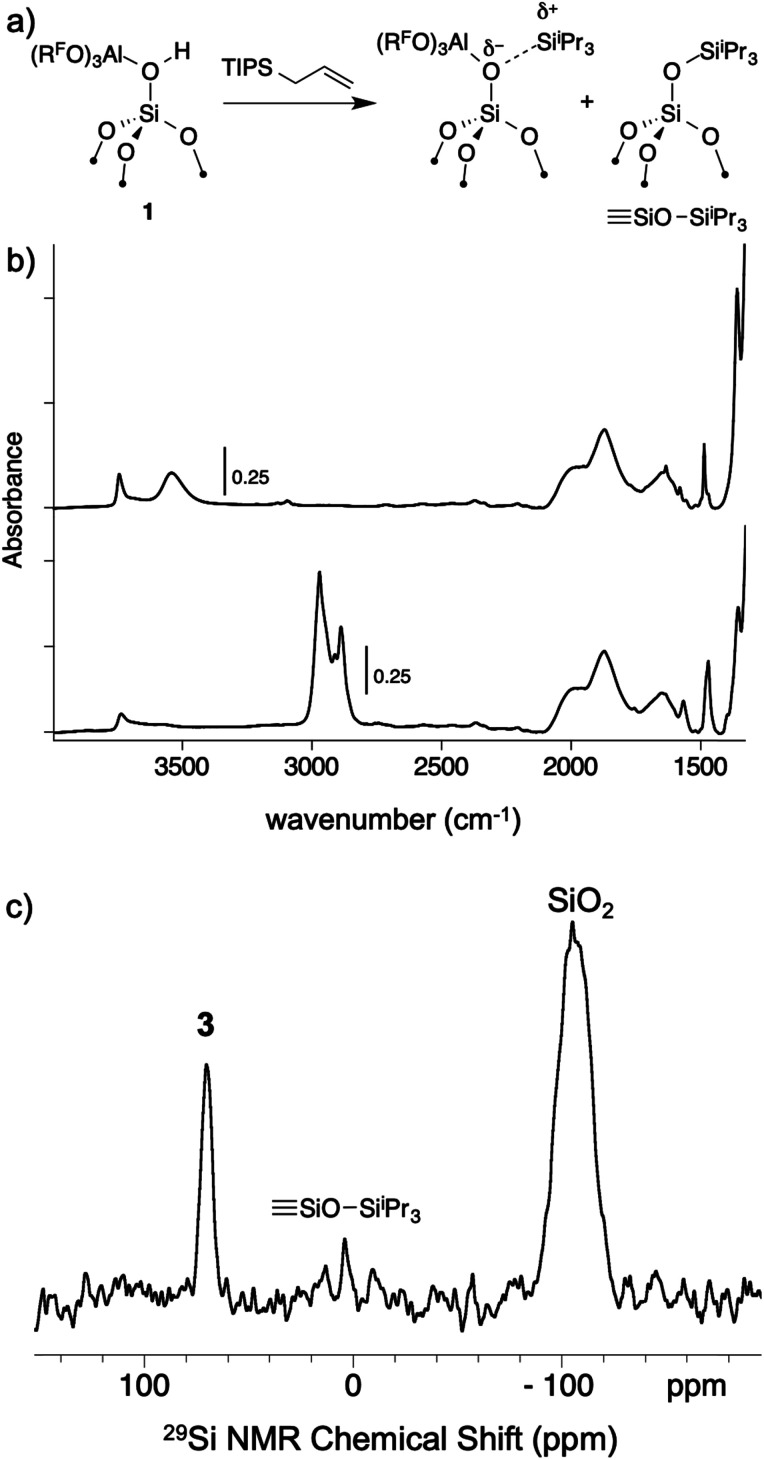
Reactivity of **1** with allyltriisopropylsilane to form **3** and small amounts of ≡SiOSi^i^Pr_3_ (a); FTIR spectrum of **1** (top) and **3** (bottom, b); ^29^Si CPMAS NMR spectrum of **3** (c).

As mentioned above, R_3_Si^+^ ions are not stable in the presence of first generation WCAs because these WCAs either react or bind to the silylium ions. The characteristics of **1** suggest that R_3_Si^+^ species may be stable on this surface. The reaction of allyltriisopropylsilane and **1** results in the formation of [^i^Pr_3_Si][(R^F^O)_3_Al–OSi≡] and small amounts of ≡SiOSi^i^Pr_3_ (**3**, Fig. 7a). The FTIR of **3** lacks the strong *ν*_OH_ for the bridging silanol observed in **1** (Fig. 7b). The ^29^Si CPMAS NMR spectrum of **3** contains a minor signal at 4.0 ppm, which is commonly observed in alkylsilane functionalized silica, and is consistent with the formation of ≡SiOSi^i^Pr_3_. The major signal in the ^29^Si CPMAS NMR spectrum is at 70 ppm (Fig. 7c), and is assigned to **3**. This chemical shift is typical of R_3_Si^+^ fragments interacting with weak ligands. The ^29^Si chemical shift of [^i^Pr_3_Si(SO_2_)][CH_6_B_11_Br_6_] appears at 85 ppm,^56^ and [Et_3_Si(toluene)][B(C_6_F_5_)_4_] appears at 94 ppm. Solvents that form stronger complexes with R_3_Si^+^ fragments appear at lower chemical shift values. For example, the ^29^Si chemical shift of [^*t*^Bu_3_Si(OH_2_)][CH_6_B_11_Br_6_] is 46.7 ppm,^57^ and [^i^Pr_3_Si(NCCH_3_)] [CH_6_B_11_Br_6_] appears at 37.2 ppm.^58^ These results suggest that the ^i^Pr_3_Si^+^ fragment in **3** is bound to a weaker ligand than MeCN or H_2_O, but a stronger ligand than toluene or SO_2_.

**Fig. 8:**
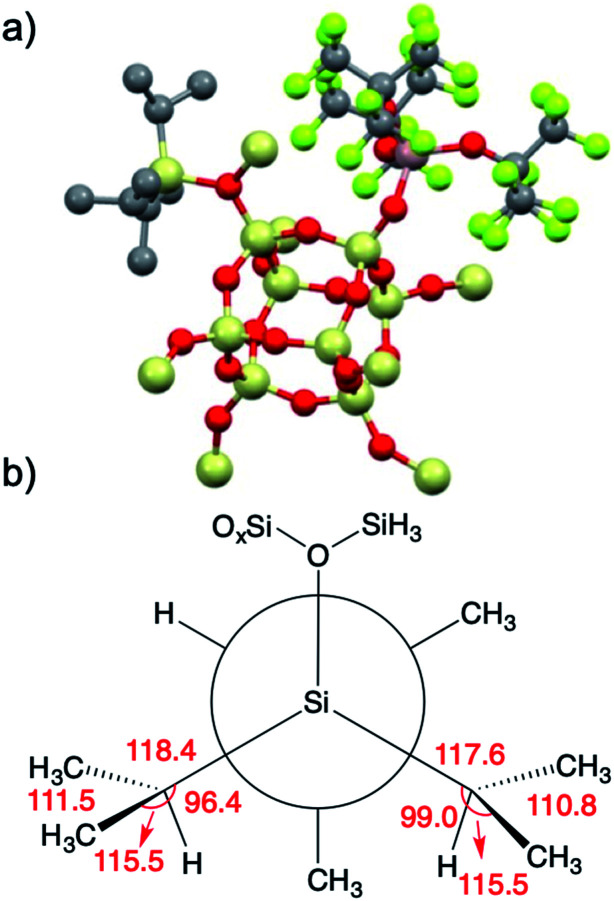
**3-DFT** with hydrogens hidden for clarity (a); Newman projection of the ^i^Pr_3_Si^+^ fragment in **3** (b). Selected angles (deg) are given in red and referred to in the text.

^i^Pr_3_Si^+^ salts contain *σ*_C–H_/3p hyperconjugation interactions between the methine C–H group of an isopropyl group and the empty 3p_*z*_ hybrid orbital on Si.^59^ The presence of hyperconjugation results in bond angles that deviate from those expected for sp^3^ geometries. A Newman projection showing the [^i^Pr_3_Si] fragment in **3** is shown in Fig. 8b. The Si–C–H bond angles in two of the ^i^Pr units are 96.4° and 99.0°, respectively. These values are lower than the expected 109.5° expected for sp^3^ carbon, and is suggestive of *σ*_C–H_/3p hyperconjugative interactions in **3-DFT**. The sum of bond angles around these isopropyl carbons (Σ_C–C–X_; X = C or Si) are 345.4° and 343.9°, respectively. Similar trends in bond angles were observed in the solid-state structure of [^i^Pr_3_Si][CH_6_B_11_Br_6_].^59^ The third isopropyl has bond angles closer to those expected for sp^3^ carbon (Si–C–H = 103.4°; Σ_C–C–X_ = 339°).

The structure of **3** was studied using DFT methods. The optimized structure of the [^i^Pr_3_Si][(R^F^O)_3_Al–OSi≡] ion pair (**3-DFT**) at the B3LYP/6-31G(d,p) level of theory is shown in Fig. 8. The calculated ^29^Si NMR chemical shift of **3-DFT** at the M06L/Al(6-311G(d,p)),6-31G(d,p) level of theory is 67 ppm, in good agreement with experimental data. The ^i^Pr_3_Si^+^ fragment coordinates to the most sterically open ≡Si–O–Si≡ bridge in the polysesquisiloxane model, and does not interact with the C–F bonds on the anionic (R^F^O)_3_Al–OSi≡ fragment. The Si–O distance in **3-DFT** is 1.86 Å, which is ∼0.1 Å longer than the Si–O bond in [^*t*^Bu_3_Si(OH_2_)][CH_6_B_11_Br_6_].^57^ The Si is displaced from the plane defined by the three carbon atoms by 0.57 Å, a larger value than typically observed for silylium ions containing carborane anions (∼0.3–0.4 Å).

**Fig. 9:**
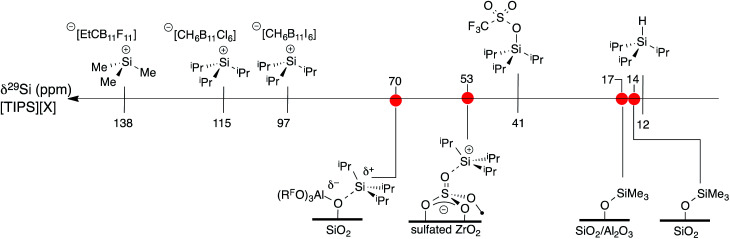
A scale relating buildup of positive charge on silicon to ^29^Si NMR chemical shift for various WCAs and oxides.

A scale of ^29^Si NMR chemical shift for selected ^i^Pr_3_Si–X species is shown in Fig. 9. The ^29^Si NMR chemical shift of triisopropylsilane is 11 ppm, while triisopropylsilyltriflate has a chemical shift of 41 ppm. R_3_Si^+^ salts containing carborane anions are more deshielded with respect to these species, appearing between 97 ppm for [^i^Pr_3_Si][CH_6_B_11_I_6_] and 115 ppm for [^i^Pr_3_Si][CH_6_B_11_Cl_6_].^60^ [Me_3_Si][EtCB_11_F_11_] contains a more weakly coordinating carborane anion and has a ^29^Si NMR chemical shift of 138 ppm,^61^ similar to silylium zwitterions.^62^

Typical ^29^Si NMR chemical shifts for alkylsilanes on oxides are also included in Fig. 9. The ^29^Si CPMAS NMR spectrum of partially dehydroxylated silica containing –OSiMe_3_ groups contains a ^29^Si NMR signal at 14 ppm.^12–15^ The ^29^Si NMR chemical shift of trimethylsilyl functionalized zeolites appear at 17 ppm.^63^ These results are inconsistent with a silylium character in these materials. To the best of our knowledge, the only [R_3_Si][oxide] type species is ^i^Pr_3_Si^+^ supported on sulfated zirconia (*δ*^29^Si = 53 ppm).^64^ The ^29^Si NMR chemical shift of **3** is 17 ppm more downfield than that of [^i^Pr_3_Si][sulfated zirconia].

The data in Fig. 9 indicates that the isotropic ^29^Si NMR chemical shift of R_3_Si–X relate to the electronics at silicon.^60^ A clear comparison is ^i^Pr_3_Si–OTf (*δ*^29^Si = 41 ppm) and [^i^Pr_3_Si][CH_6_B_11_Cl_6_] (*δ*^29^Si = 115 ppm). Triflate anions bind to ^i^Pr_3_Si fragments stronger than electron deficient carborane anions, which modulates the Lewis acidity of the ^i^Pr_3_Si-fragment in these compounds because silicon is more positively charged in carborane salts than triflates. This is also reflected in the geometry of the ^i^Pr_3_Si-fragment, which becomes more planar in carborane salts than typical sp^3^ organosilanes. Less clear was if this trend would also apply to alkylsilanes supported on oxides. The available ^29^Si chemical shift values for R_3_Si-supported on silica and silica–alumina suggested that alkylsilanes do not form R_3_Si^+^ sites.^12–15,63^ This is a result of formation of ≡SiO–SiR_3_ sites on these material surfaces.

Sulfated zirconium oxide and **1** are more acidic than silica or silica alumina based on proton affinity calculations.^33^ This suggests that these ^i^Pr_3_Si-functionalized materials would contain ^29^Si NMR chemical shifts more downfield than R_3_Si-functionalized silica or silica alumina. The ^29^Si chemical shift of [^i^Pr_3_Si][sulfated zirconia] (*δ*^29^Si = 53 ppm) and **3** (*δ*^29^Si = 70 ppm) are consistent with formation of species with R_3_Si^+^ character. However, these chemical shifts are far from those of ^i^Pr_3_Si^+^ carborane salts. These data indicate that ^29^Si NMR chemical shift trends for molecular R_3_Si–X also apply to surface species. This implies that the ^29^Si NMR chemical shift on R_3_Si-functionalized surfaces gives information about ion-pairing on surfaces sites, which could be important in designing catalytic sites on these weakly coordinating surfaces.

Silylium ions are strong Lewis acids that catalyze or mediate numerous chemical reactions.^65–67^ Silylium ions activate C–F bonds to form R_3_Si–F and carbocation intermediates,^68–72^ which are rapidly quenched in the presence of excess silane to form C–H bonds. **3** activates C–F bonds in 1-adamantylfluoride in the presence of Et_3_SiH at 0 °C to give adamantane (TON = 18). This reactivity is consistent with silylium character in the ^i^Pr_3_Si^+^ fragment in **3**. However, **3** is less stable than ^i^Pr_3_Si^+^ sites supported on sulfated zirconia, which gives 160 turnovers in this reaction.^64^ Solution ^19^F NMR spectra monitored during the C–F bond activation reaction contain signals for Al(OR^F^)_3_ and HOC(CF_3_)_3_, indicating that the low stability of **3** is probably related to decomposition reactions of the surface aluminum anion under these conditions (Fig. S20†).

## Conclusions

This study shows that design strategies for WCAs in solution can be applied to generate well-defined surface WCAs. The reaction of SiO_2-700_ with Al(OR^F^)_3_*PhF in perfluorohexane forms ≡Si–OH⋯Al(OR^F^)_3_ (**1**) and contains strong Brønsted acid sites based on GPA calculations. Experimental evaluation of the *ν*_NH_ stretch in [Oct_3_NH][(R^F^O)_3_Al–OSi≡)] (**2**) shows that this material forms weaker ion-pairs than typical first generation WCAs. **1** reacts with allyltriisopropylsilane to generate [^i^Pr_3_Si][(R^F^O)_3_AlOSi≡] (**3**), a rare example of a silylium-like Lewis acid supported on an oxide, and to the best of our knowledge the only example supported on derivatized silica. Though the *ν*_NH_ stretch of **2** and ^29^Si NMR chemical shift of **3** show that **1** does fulfill the prerequisites to form WCAs, these data also suggest that ion-pairing on these surface sites is stronger than carborane or [B(C_6_F_5_)_4_] anions. Neutral Lewis acids stronger than Al(OR^F^)_3_ are necessary to form strong Brønsted acids with partially dehydroxylated silica to form weaker coordinating anions than **1**.

## Conflicts of interest

There are no conflicts to declare.

## Supplementary Material

SC-011-C9SC05904K-s001
