# Supplementary material for: Al(ORF)3 (RF = C(CF3)3) activated silica: a well-defined weakly coordinating surface anion
Source: Chem Sci. 2019 Dec 19;11(6):1510–7. doi: 10.1039/c9sc05904k (PMC8148071; doi:10.1039/c9sc05904k)
Supplement: SC-011-C9SC05904K-s001 [file SC-011-C9SC05904K-s001.pdf]

Electronic Supplementary Material (ESI) for Chemical Science.
This journal is © The Royal Society of Chemistry 2019

Supplementary information

$Al(OR^F)_3$ ($R^F = C(CF_3)_3$) Activated Silica: A Well-Defined Weakly Coordinating Surface Anion

Damien B. Culver,[1] Amrit Venkatesh,[2] Winn Huynh,[1] Aaron J. Rossini,[2] Matthew P. Conley[1]*

[1]Department of Chemistry, University of California, Riverside, California 92507, United States

[2]Department of Chemistry, Iowa State University, Ames, Iowa 50011, United States

Email: matthew.conley@ucr.edu

Table of Contents

1. **General Considerations S2**
2. **Synthesis and characterization of 1-3 .... S3**
    a. **1 .... S3**
    b. **2 .... S8**
    c. **3 .... S11**
3. **^{27}Al NMR simulation parameters for 1-3 .... S14**
4. **Leaching experiment S17**
5. **Hydrodefluorination of adamantyl fluoride S18**
6. **Computational details S19**
7. **References S38**

General Considerations

All manipulations were performed under an inert atmosphere of nitrogen or argon using standard glovebox, schlenk or high vacuum techniques. Grafting reactions were performed in double schlenk flasks or flasks equipped with teflon valves that connect directly to high vacuum lines.[1] Cyclohexane-D12, benzene-D6, acetonitrile-D3, and methylene chloride-D2 were purchased from Cambridge Isotope laboratories. Cyclohexane was dried over sodium/benzophenone, degassed and distilled under vacuum. Pentane was dried over tetraglyme/sodium/benzophenone, degassed and distilled under vacuum. Perfluorohexane, fluorobenzene, trioctylamine, and triethylsilane were dried over CaH_2, degassed, and vacuum distilled prior to use. Allyltriisopropylsilane was stored over activated molecular sieves and degassed prior to use. $SiO_{2\text{-}700}$[2], and PhF-Al[OC(CF_3)$_3$]$_3$[3] were synthesized as previously reported.

FT-IR spectra were recorded as pressed pellets using a Bruker Alpha IR spectrometer in an argon-filled glovebox. Aluminum elemental analyses were carried out by digesting solid samples in dilute nitric acid and measuring the samples at the University of California, Riverside Environmental Sciences Research Laboratory (ESRL) on a Perkin – Elmer Optima 7300DV ICP – OES. Fluorine and CHN analyses were performed by the microanalysis laboratory at the University of Illinios, Urbana – Champagne.

NMR Spectroscopy

Solution NMR spectra at 7.05 T were acquired out on an Avance Bruker 300. ^{1}H NMR spectra were referenced to the NMR solvent residual peak. Solution ^{19}F{^{1}H} spectroscopy were referenced to an external standard of C_6F_6 (-163.9 ppm). Solid state NMR spectra at UC Riverside were recorded in 4 mm zirconia rotors at 8 – 12 KHz spinning at the magic angle at 14.1 T on an Avance Bruker NEO600 spectrometer equipped with a standard-bore magnet. Solid state ^{19}F MAS NMR at the California Institute of Technology were recorded at 11.7 T in a 4 mm rotor spinning at 10 kHz on an Avance Bruker 500.

Solid-state NMR experiments at 9.4 T at Iowa State University were performed on a Bruker Avance III HD spectrometer equipped with wide-bore magnet. Experiments were performed at an MAS frequency (ν_{rot}) of 25 kHz using a 2.5 mm triple-resonance probe. 1D ^{1}H NMR spectra were acquired using the DEPTH pulse sequence[4] comprising of a 90° excitation pulse and followed by two successive 180° pulses for background suppression at 100 kHz radiofrequency (RF) field.

The ^{1}H{^{27}Al} RESPDOR[5-6] experiment was performed with the $SR4_1^2$ dipolar recoupling sequence[7] on the ^{1}H channel applied with a radiofrequency (RF) field of twice the MAS frequency ($2 \times \nu_{rot}$). The saturation pulse was applied on the ^{27}Al channel at 80 kHz RF field with a duration of 60 μs ($1.5 \times \tau_{rot}$, $\tau_{rot} = 1/\nu_{rot}$). The experiment was performed in an interleaved manner where a control dataset is obtained without the pulse on the ^{27}Al channel for every recoupling duration. Numerical simulations of ^{1}H-^{27}Al RESPDOR were performed with SIMPSON *v*4.2.1[8-10]. The ^{1}H{^{27}Al} RESPDOR curve shown in the main text compares $\Delta S/S_0$ with numerical simulations performed with a saturation factor (*f*) =0.55 and different ^{1}H-^{27}Al dipolar coupling constants/inter-nuclear distances. The curve corresponding to a ^{1}H-^{27}Al distance of 2.46 Å shows the best agreement with experiment, consistent with the DFT calculated structure of **1**. Numerical simulations were performed in SIMPSON with the start operator set to I_{1x} and the detect operator set to I_{1p}. Powder averaging was performed using the 'rep320' crystallite orientation file

comprising of 320 (α, β) pairs. 16 γ angles were used. An ideal ^{1}H 180° pulse was used, whereas the ^{27}Al saturation pulse used an 80 kHz RF field and a duration of 60 μs ($1.5 \times \tau_{rot}$) to mimic experimental conditions. The ^{27}Al C_Q and η were set to 15.2 MHz and 0.0, respectively. The relative orientations (Euler angles) of the ^{27}Al quadrupole and CSA tensors and the ^{1}H-^{27}Al dipole vector were set according to the DFT optimized structure of **1**.

The proton detected $^{27}Al \rightarrow ^{1}H$ D-RINEPT experiment[11-12] was performed with a 0.1 s recycle delay, 4096 scans, 100 kHz indirect spectral width and 92 t_1 increments. The STATES-TPPI procedure was used to achieve sign discrimination and obtain absorptive peaks in the indirect dimension. Rotor synchronized $SR4_1^2$ dipolar recoupling was applied on the ^{1}H channel with RF set to $2 \times \nu_{rot}$. 4 μs central transition (CT) selective 90° pulses were applied on the ^{27}Al channel. RAPT pulses[13]were applied on the ^{27}Al channel prior to the D-RINEPT transfer step using 38 μs frequency switched WURST (wideband, uniform rate, smooth truncation) pulses separated by a 2 μs delays at 31 kHz RF field.

All solid-state NMR were processed using Topspin *v*3.6.1. ^{27}Al analytical simulations were performed using ssNake v1.1.[14]

Synthesis and characterization of 1 – 3

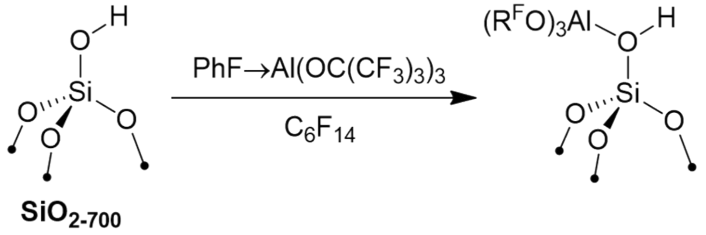

Synthesis of 1: SiO_{2-700} (2 g, 0.52 mmol OH) and PhF-Al[OC(CF_3)$_3$]$_3$] (480 mg, 0.58 mmol) were transferred to one arm of a double-Schlenk flask inside an argon-filled glovebox. Perfluorohexane (ca. 10 ml) was transferred under vacuum to the flask at 77 K. The mixture was warmed to room temperature and gently stirred for two hours. The clear solution was filtered to the other side of the double Schlenk. The remaining solid was washed by condensing solvent from the other arm of the double Schlenk at 77 K, warming to room temperature, stirring for 2 minutes, and filtering the solvent back to the other side of the flask. This was repeated two times. The solid was dried under diffusion pump vacuum for 1 hour. The white material was stored in a glovebox freezer at -20 °C. FTIR: $\nu_{O\text{-}H}$ = 3743 (≡Si–O**H**) and 3542 (≡Si–O**H**---Al(OR^F)$_3$) cm^{-1}. Solid state NMR: ^{1}H MAS NMR (600 MHz): 7.1 (PhF), 4.9 (≡Si–O**H**---Al(OR^F)$_3$), 2.3 (≡Si–O**H**); ^{19}F MAS NMR (470 MHz): -78.5 (AlOC(C**F**$_3$)$_3$), -134 (Ph**F**); $^{13}C\{^{1}H\}$ MAS NMR (151 MHz): 121 (q, $^{1}J_{C\text{-}F}$: 277 Hz, --OC(**C**F$_3$)$_3$), 78 (-O**C**(CF$_3$)$_3$); $^{29}Si\{^{1}H\}$ NMR (119 MHz): -95 (≡**Si**–OH---Al(OR^F)$_3$) and -104 (**Si**O$_2$) ppm. Elemental analysis: 0.64 % Al, 2.52 % C, and 7.61 % F

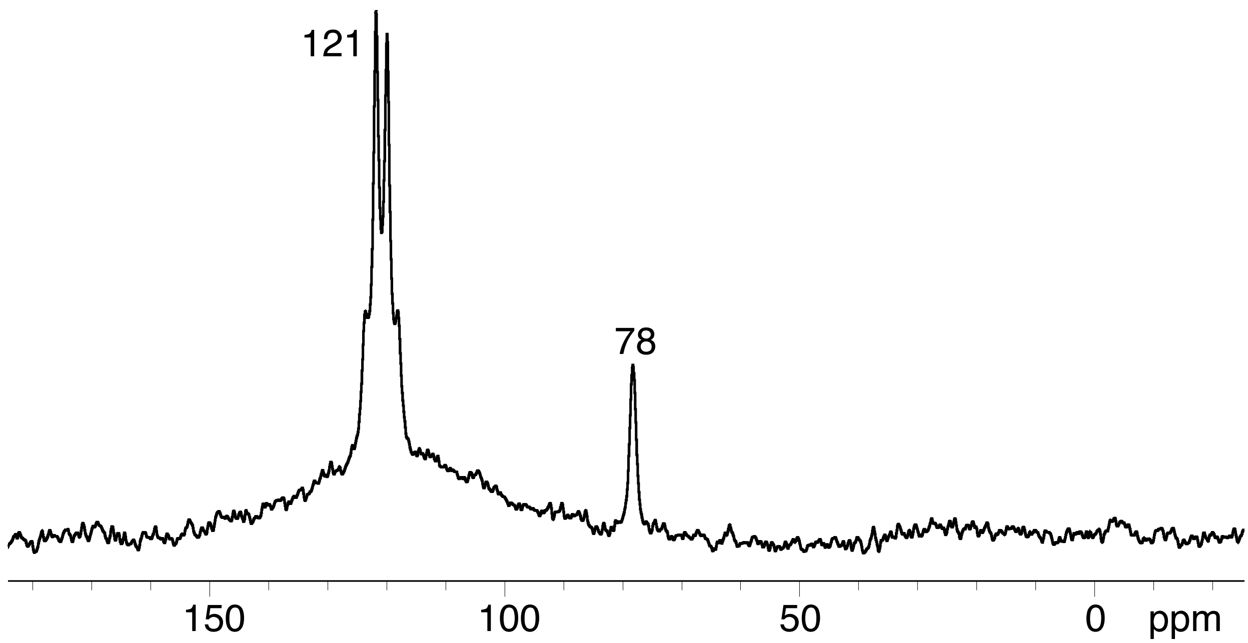

Figure S1. $^{13}C\{^{1}H\}$ HP-DEC MAS NMR spectrum of **1** spinning at 10 kHz, relaxation delay of 3 seconds (the broad peak centered around 120 ppm is rotor background).

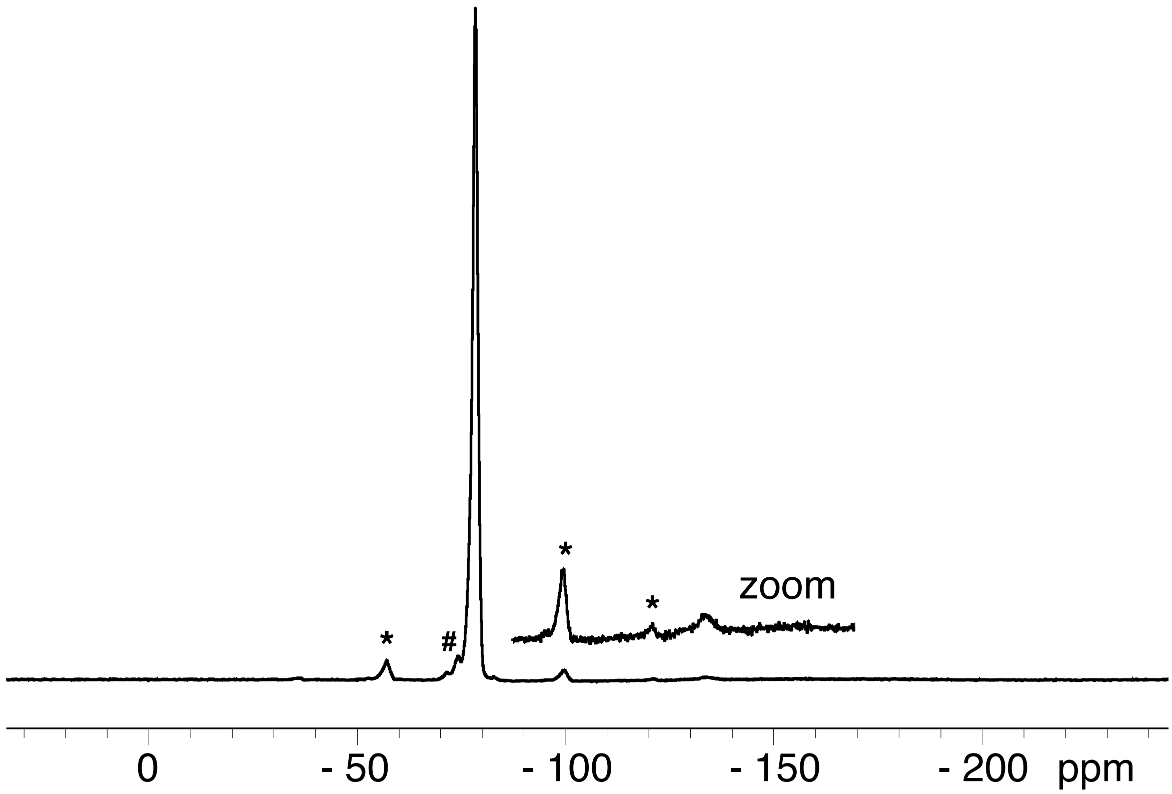

Figure S2. ^{19}F NMR spectrum of **1** spinning at 10 kHz, * = spinning sideband. The zoom contains the signal for PhF at -134 ppm, # = $HOC(CF_3)_3$ due to thermal decomposition.

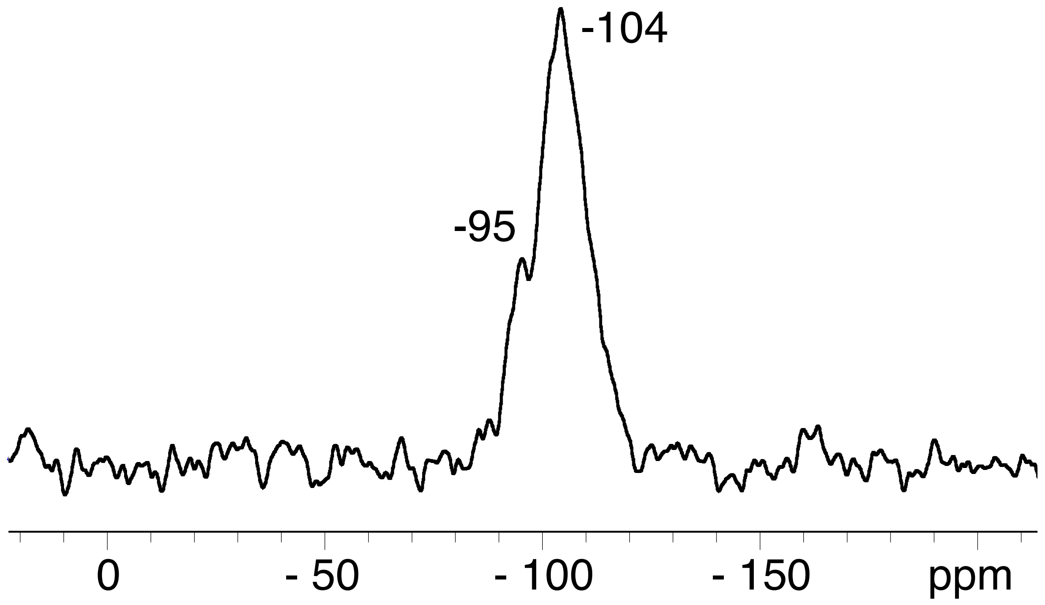

Figure S3. ^{29}Si CP-MAS NMR spectrum of **1** spinning at 8 kHz.

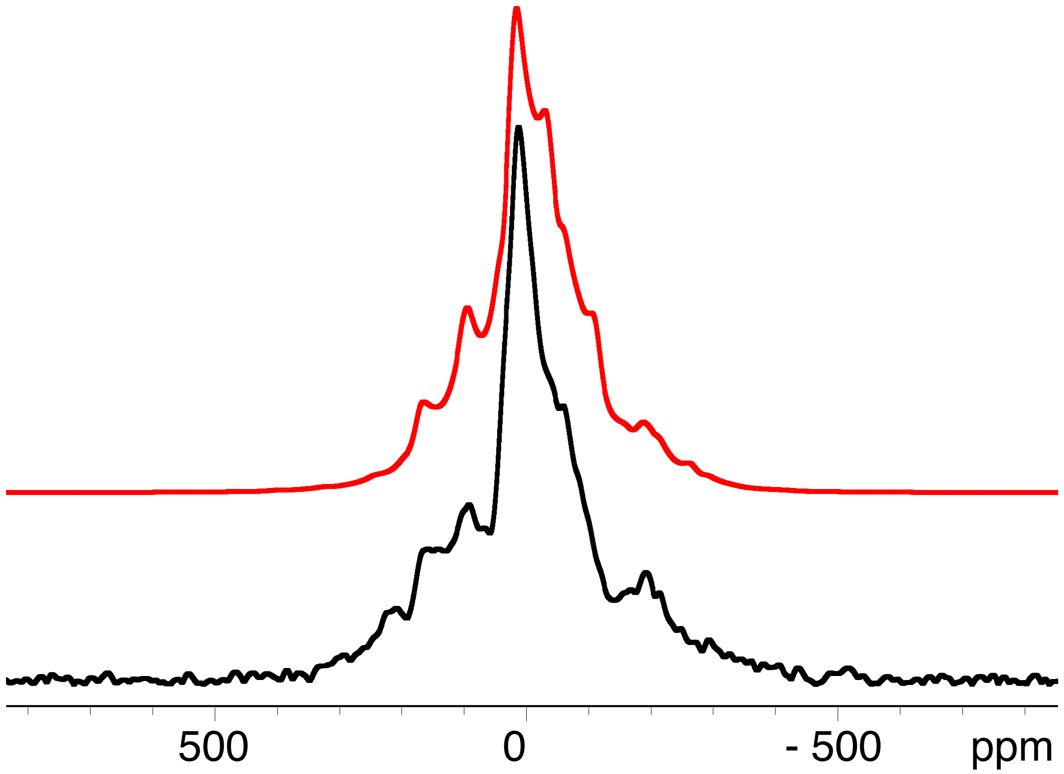

Figure S4. $^{27}Al\{^{1}H\}$ MAS NMR spectrum of 1 spinning at 12 kHz (black) and simulation (red).

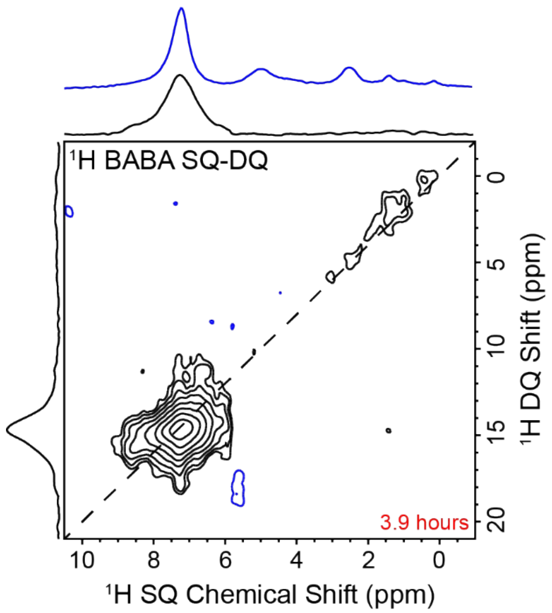

Figure S5. 2D ^{1}H single quantum (SQ)-double quantum (DQ) experiment performed using the back-to-back (BABA) recoupling sequence[15] at 25 kHz MAS and 9.4 T. A single rotor cycle of BABA recoupling was used at 100 kHz radiofrequency (RF) field during the excitation and reconversion periods. An overlay of the 1D ^{1}H NMR experiment is shown above the ^{1}H projection of the 2D spectrum (blue).

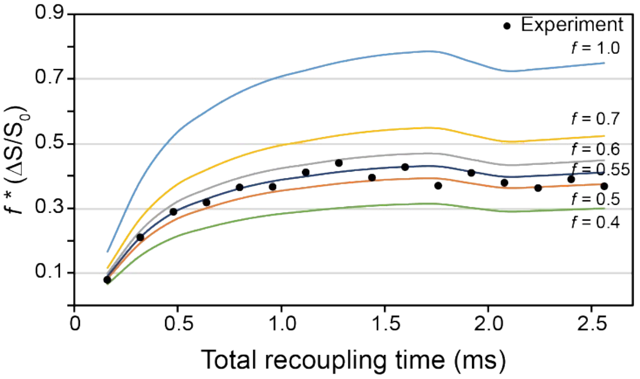

Figure S6. Plot comparing ^{1}H{^{27}Al} RESPDOR experimental and numerically simulated dephasing intensities as a function of total $SR4_1^2$ recoupling time. Comparison of experimental $\Delta S/S_0$ RESPDOR curves with numerical simulations performed with different saturation factor (*f*) for a fixed ^{1}H-^{27}Al dipolar coupling constant of 2.1 kHz (2.46 Å ^{1}H-^{27}Al distance). The saturation factor *f* was applied as a scaling factor to the calculated RESPDOR curves ($f \times \Delta S/S_0$) to account for incomplete saturation of ^{27}Al satellite transitions and/or additional ^{1}H signal intensity attributed to ^{1}H spins that are isolated from ^{27}Al (see reference 6).

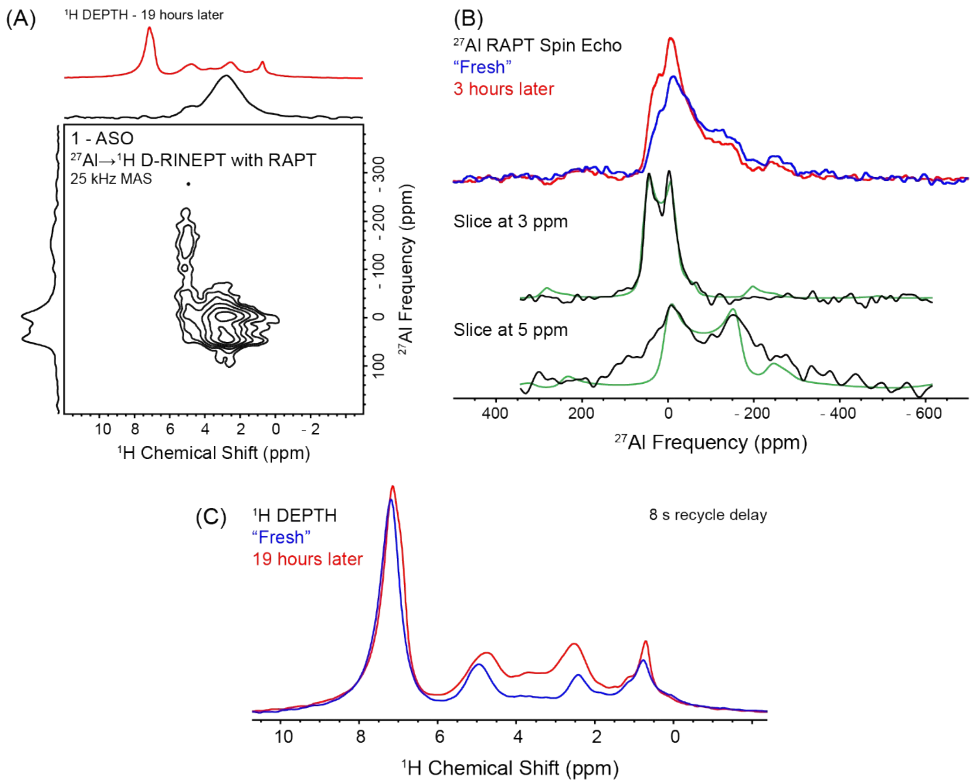

Figure S7. (A) 2D ^{27}Al→^{1}H RAPT D-RINEPT spectrum acquired with the $SR4_1^2$ dipolar recoupling sequence at 25 kHz MAS and 9.4 T (20.5 hours experiment time). (B) Comparison of ^{27}Al RAPT spin echo spectra, ^{27}Al slices (black) extracted from the 2D D-RINEPT spectrum at ^{1}H chemical shifts of 3 and 5 ppm and simulated spectra (green). The RAPT spin echo spectra shown were acquired one after the other. (C) Comparison of 1D ^{1}H DEPTH spectra acquired initially and 19 hours later. During this time, the rotor was spinning under N_2 gas.

The 2D ^{27}Al→^{1}H D-RINEPT spectrum shows that the acidic proton at 5.0 ppm correlates with a broad ^{27}Al NMR signal at 50 ppm (C_Q = 15.7 MHz), which is assigned to **1**. The observed C_Q of this site is consistent with the 14.1 T measurements shown in Table S1. The INEPT spectrum also shows an intense correlation between a ^{1}H NMR signal at 3.0 ppm and a sharper ^{27}Al NMR signal at 73 ppm (C_Q = 10.0 MHz). This signal is assigned to a higher symmetry ^{27}Al species that forms during the course of sample rotation, most likely because of partial hydrolysis of **1** in the imperfectly sealed 2.5 mm rotors. Consistent with this interpretation, the ^{1}H DEPTH spectrum of **1** obtained immediately at the start of MAS experiments ("fresh") and after 19 hours of continuous MAS ("19 hours later") shows a clear increase in total ^{1}H integrated signal intensity, suggesting ingress of water into the rotor (Figure S6C). The ^{27}Al RAPT spin echo spectrum of the "fresh" sample was obtained immediately after starting MAS, however, acquisition of the spectrum required ca. 3 hours, during which partial hydrolysis likely occurred (Figure S6B). A second ^{27}Al spin echo spectrum was then obtained ("3 hours later") and the intensity of the broad ^{27}Al signal was observed to decrease slightly, while the narrower ^{27}Al signal increased slightly. All of these observations are again consistent with partial hydrolysis of **1** in the rotor.

ASO + n-octyl$_3$N (2): ASO (200 mg, 0.05 mmol ≡Si–OH---Al(OR^F)$_3$) was loaded into a teflon – valved flask. Pentane (2 ml) was vacuum transferred to the solid at -196 °C using a high vacuum line. In a N_2 filled glovebox, trioctylamine (12 µL, 0.03 mmol) was added to the slurry. The reaction was gently stirred for 30 minutes then the solution was removed by cannula under argon flow. The solid was washed 2 X more by vacuum transferring in more pentane (2 mL) then removing solvent by cannula again. The cream colored solid was dried under vacuum. FTIR: 3070 cm^{-1} (N-H). Solid state NMR: ^{1}H NMR (600 MHz): 7.1 (PhF), 1.0 (n-octyl), 0.7 (n-octyl); ^{19}F MAS NMR (470 MHz): -77 (AlOC(C**F**$_3$)$_3$); ^{13}C{^{1}H} NMR (151 MHz): 121 (q, $^1J_{C-F}$: 277 Hz, -OC(**C**F$_3$)$_3$), 79 (-O**C**(CF$_3$)$_3$), 55 (n-octyl), 53 (n-octyl), 31 (n-octyl), 29 (n-octyl), 26 (n-octyl), 22.5 (n-octyl), 12 (n-octyl); ^{29}Si{^{1}H} NMR (119 MHz): -105 (**Si**O$_2$) ppm. Elemental analysis: 0.63 % Al, 0.26 % N, 5.83 % C and 6.5 F %

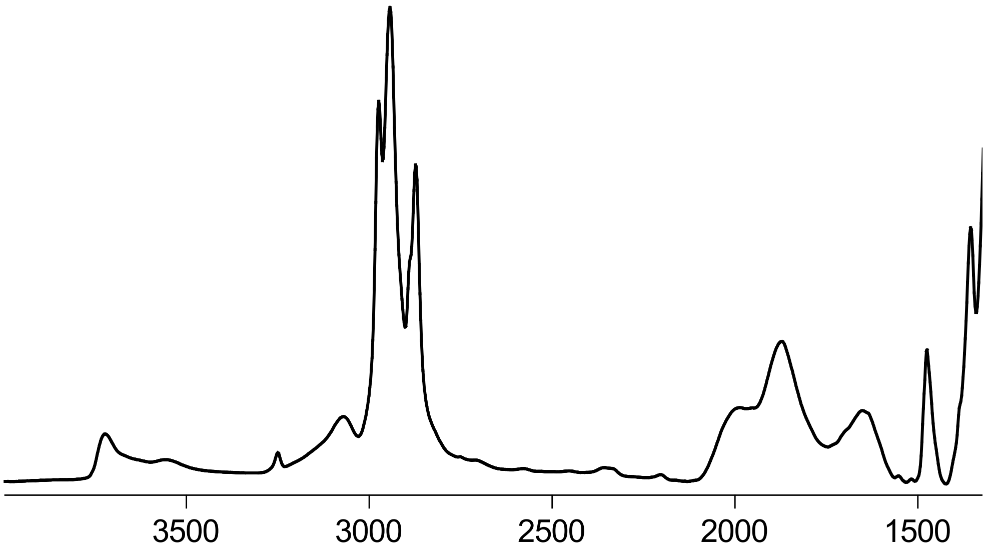

Figure S8. FTIR spectrum of **2**, wavenumbers (cm^{-1}).

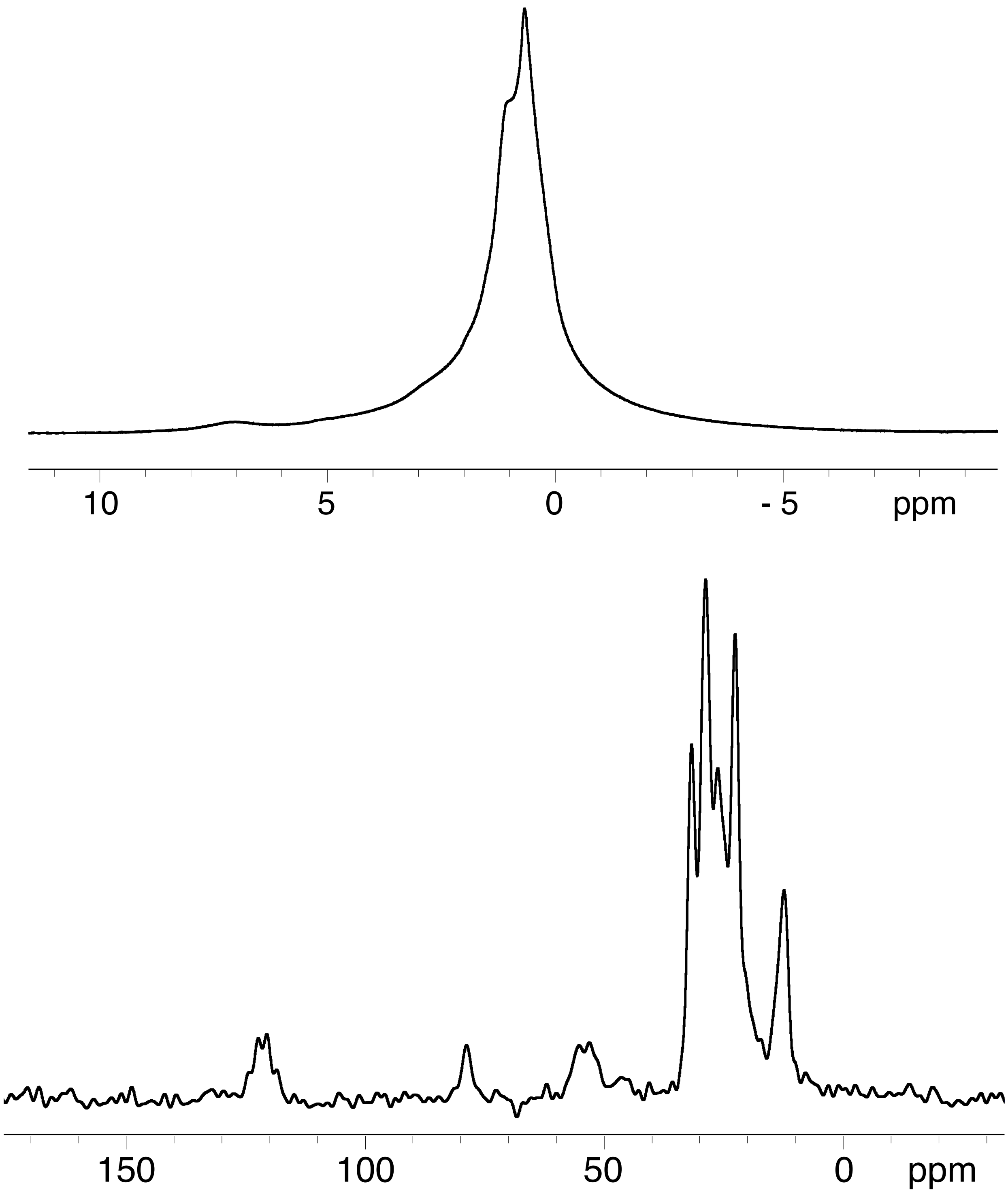

Figure S9. ^{1}H and $^{13}C\{^{1}H\}$ CP-MAS NMR spectra of **2** spinning at 10 kHz.

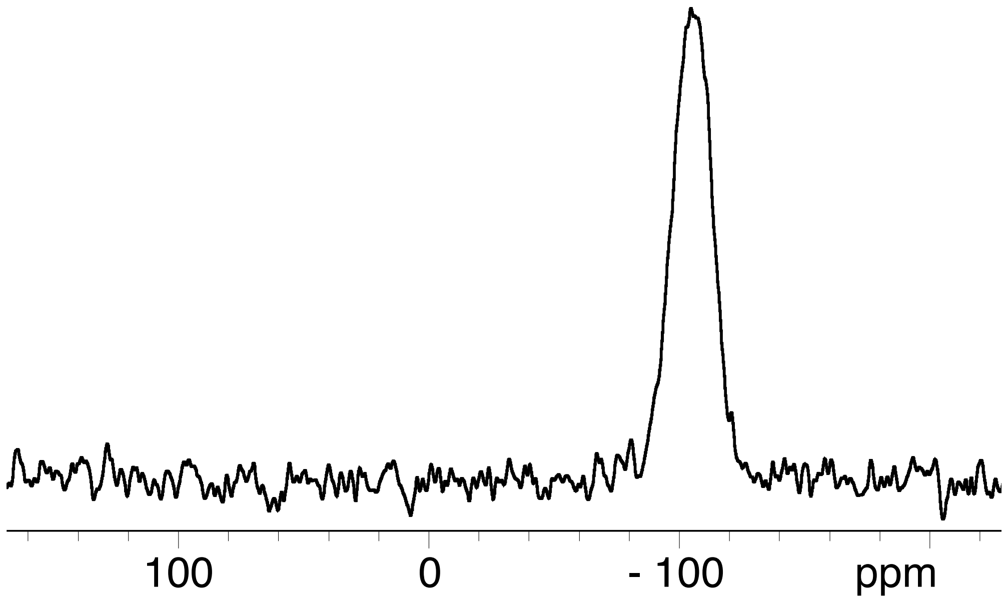

Figure S10. $^{29}Si\{^1H\}$ CP-MAS NMR spectrum of **2** spinning at 8 kHz.

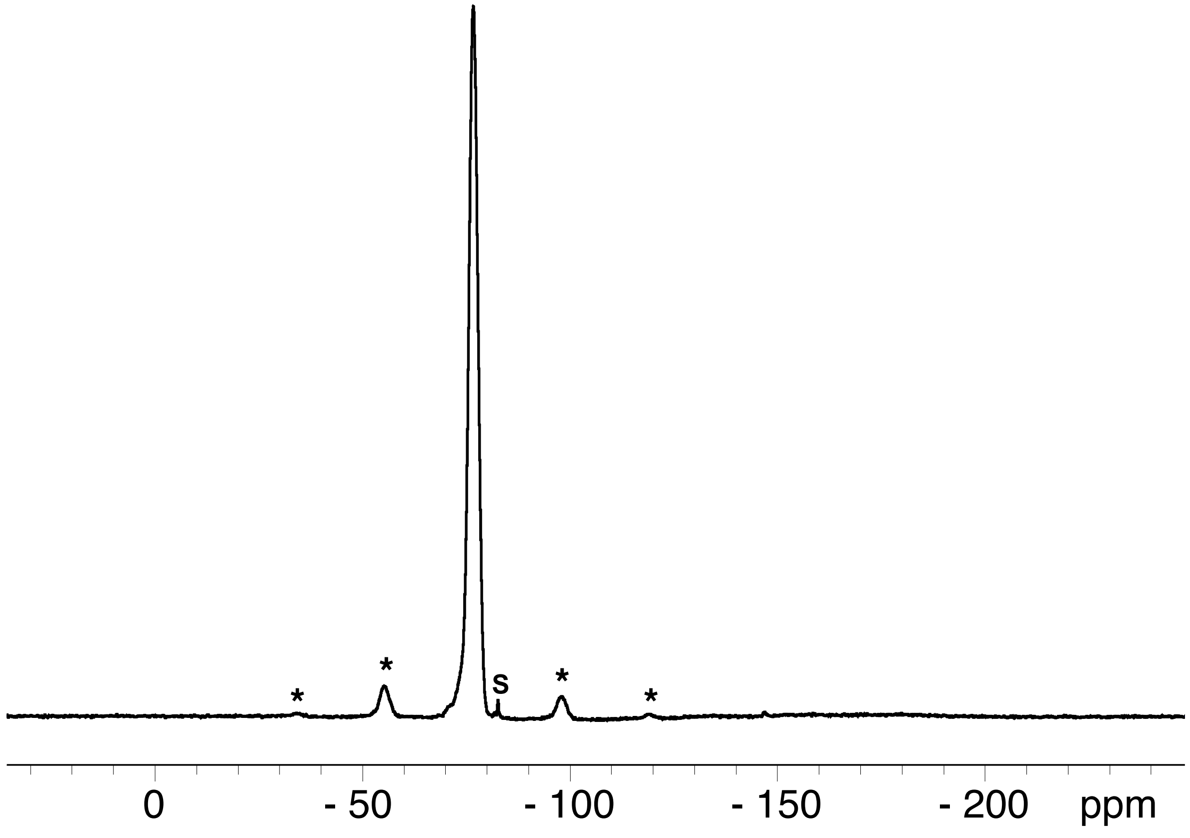

Figure S11. ^{19}F NMR spectrum of **2** spinning at 10 kHz, * = spinning sideband. S = unknown impurity

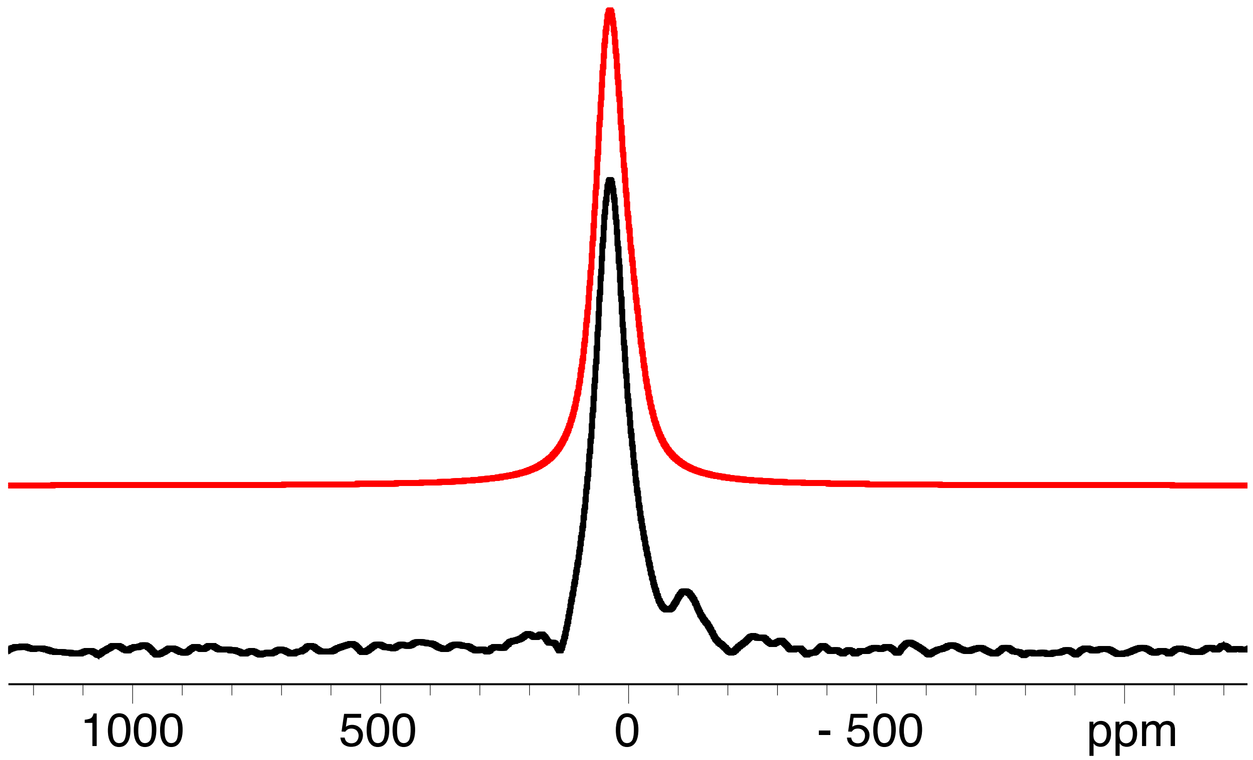

Figure S12. static $^{27}Al\{^1H\}$ solid state NMR spectrum of **2** (black), and simulation (red).

Synthesis of 3: 1 (1 g, 0.24 mmol ≡Si–OH---Al(OR^F)$_3$) was loaded into a teflon – valved flask and evacuated under diffusion pump vacuum. Pentane (~5 mL) was transferred to the flask at -196 °C. The slurry was warmed up to 0 °C and allyltriisopropylsilane (0.06 mL, 0.26 mmol) was added by syringe under the flow of argon. The slurry was stirred at 0 °C for 2 hours. The solution was decanted by cannula, washed with freshly distilled pentane (2 x 5 mL). The solid was dried under diffusion pump vacuum to give a cream colored solid **3**.

Analysis of the washings by solution ^1H NMR, after ca. 90 % of the pentane was allowed to evaporate, contains signals characteristic of oligomers derived from cationic oligomerization of propene. Control experiments show that **1** oligomerizes propene under conditions similar to formation of **3**, suggesting that propene formed in the grafting reaction is oligomerized by residual Bronsted sites under these conditions (Figure S13). Solid state NMR: ^1H NMR (600 MHz): 7.0 (PhF), 0.88 (^{i}Pr); ^{19}F MAS NMR (470 MHz): -78 (-OC(C**F**$_3$)$_3$); $^{13}C\{^1H\}$ NMR (151 MHz): 121 (-OC(**C**F$_3$)$_3$), 78 (-O**C**(CF$_3$)$_3$), 15 (SiCH(**C**H$_3$)$_2$), 12 (Si**C**H(CH$_3$)$_2$); $^{29}Si\{^1H\}$ NMR (119 MHz): 70 (≡Si–O**Si**^{i}Pr$_3$---Al(OR^F)$_3$), 4 (≡Si–O**Si**^{i}Pr$_3$) and -105 (**Si**O$_2$) ppm. Elemental analysis: 0.65 % Al, 4.24 % C, 0.47 % H and 4.5 F %.

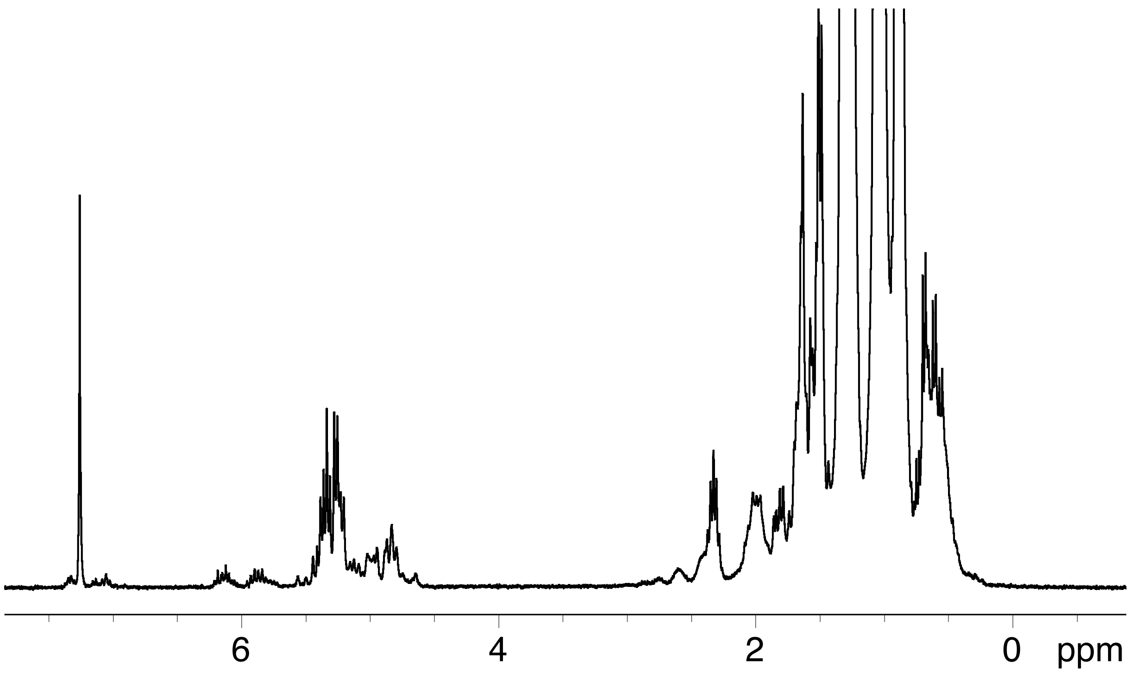

Figure S13. ^{1}H NMR spectrum of the washings after the synthesis of **3** in $CDCl_3$.

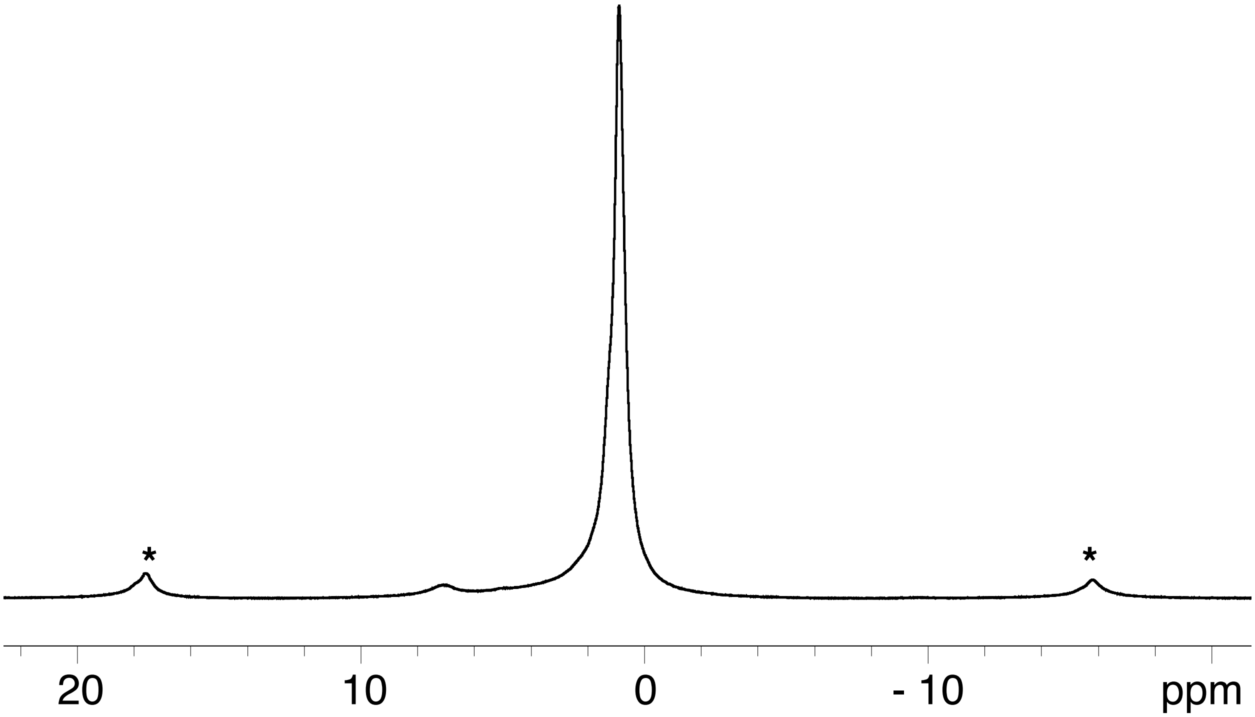

Figure S14. ^{1}H NMR spectrum of **3** spinning at 10 kHz, * = spinning sidebands.

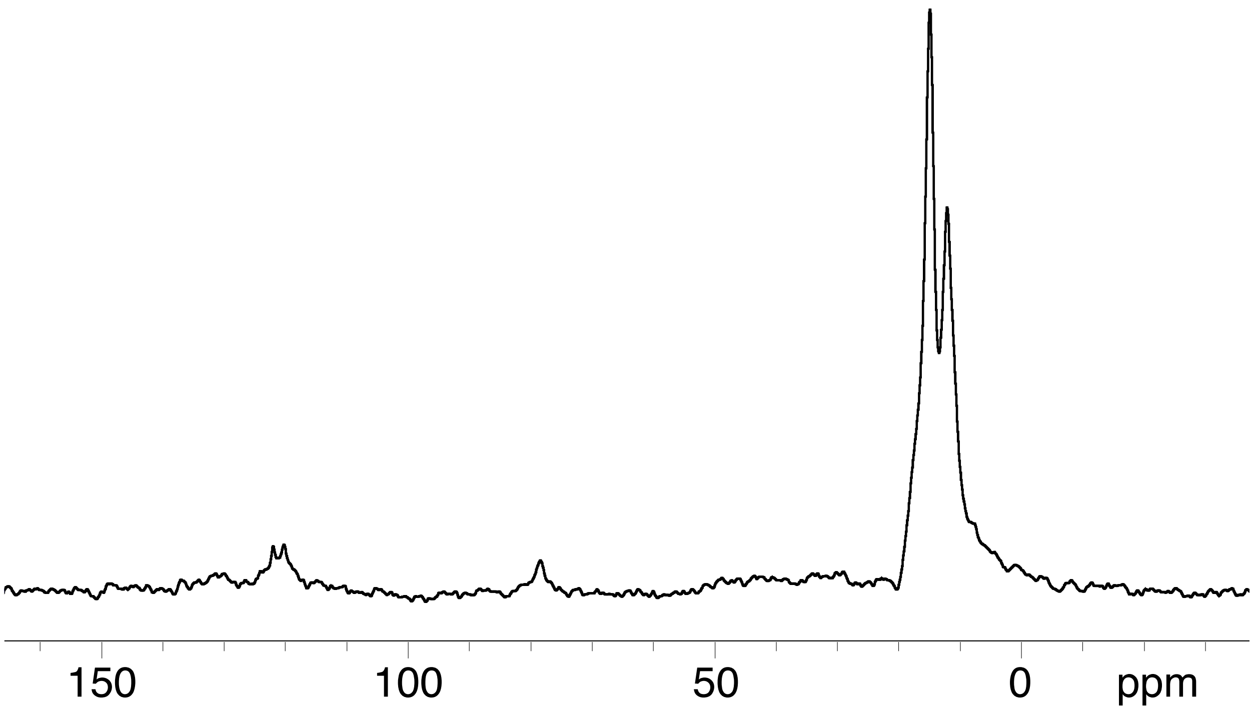

Figure S15. ^{13}C CP-MAS NMR spectrum of **3** spinning at 10 kHz.

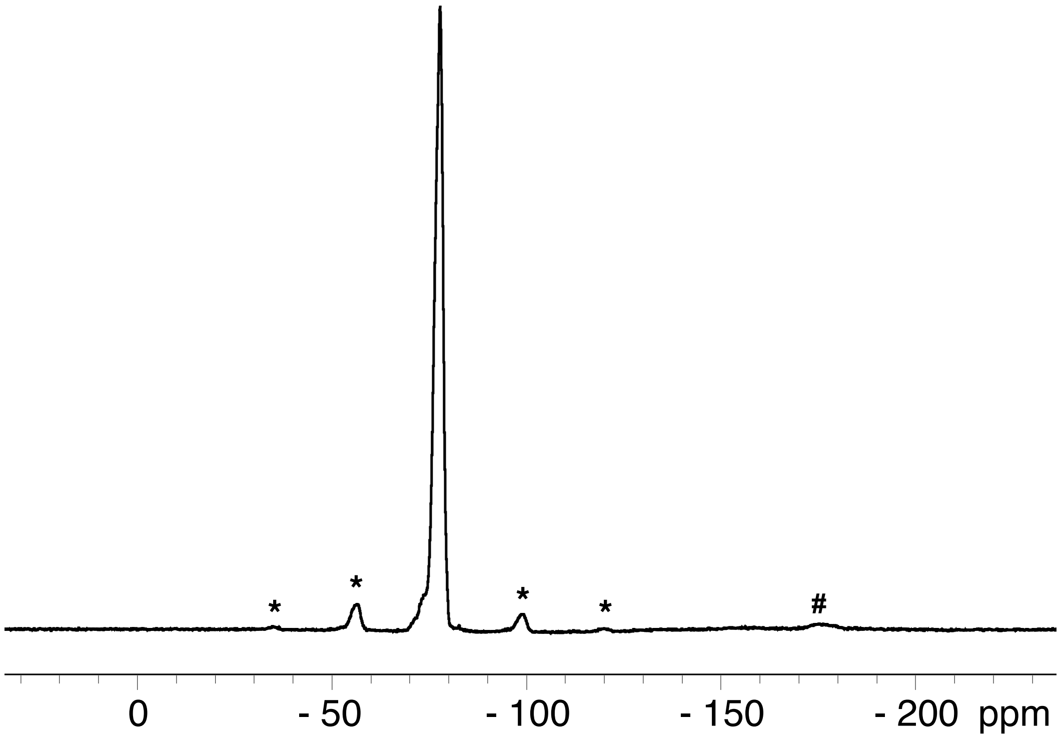

Figure S16. ^{19}F NMR spectrum of **3** spinning at 10 kHz, * = spinning sideband. # = $^{i}Pr_3SiF$ due to thermal decomposition.

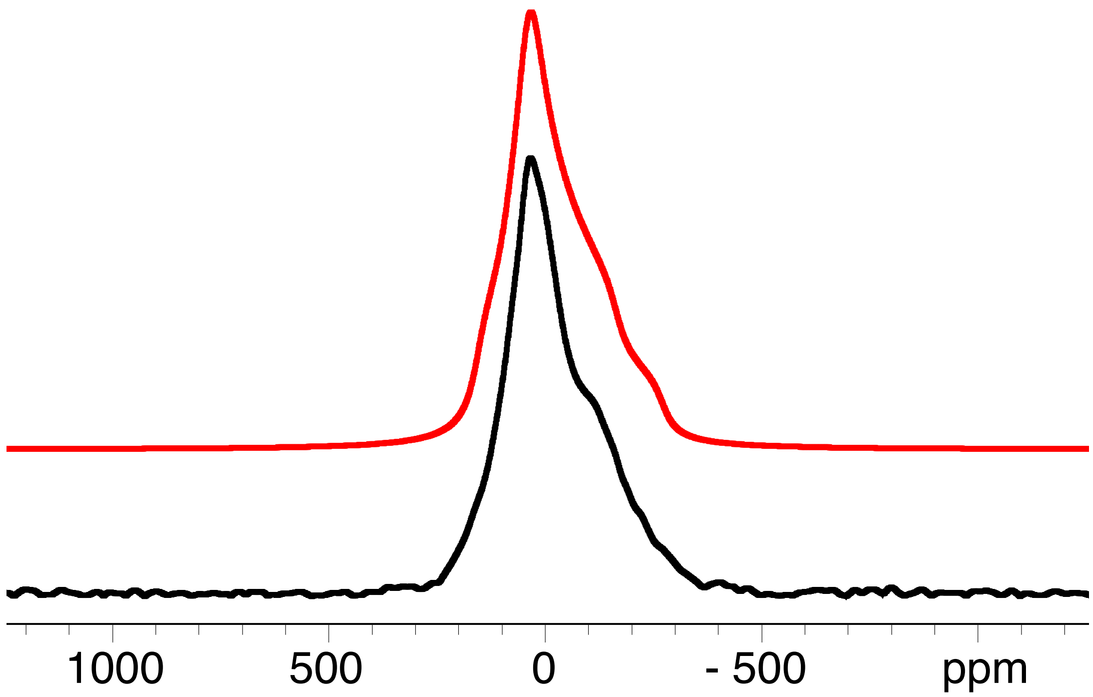

Figure S17. Static $^{27}Al\{^{1}H\}$ NMR spectrum of **3**;Top = simulation and bottom = experimental spectrum.

Table S1. ^{27}Al simulation parameters for **1** – **3**[a]

Material	δ_{iso} (ppm)	Ω (ppm)	κ	C_Q (MHz)	η	α	β	γ
**1**	43.2	196	-0.41	14.6	0.23	60	34	75
**1**[b]	43.0	227	-0.96	14.6	0.20	60	35	75
**2**	45.6	90	-0.22	6.7	0.47	332	15	173
**3**	33	118	-0.14	13.0	0.85	0	33	0

a.) taken from simulations of static spectra shown in Figures 2 (from the main text), S12, and S17; and b.) taken from the simulation of the MAS spectrum in Figure S4.

Stability of 1 in common solvents: 1 (50 mg) was loaded into a teflon – valved NMR tube then solvent (0.5 mL) was vacuum transferred over the solid. The $^{19}F\{^{1}H\}$ NMRs were recorded 1 hour after solvent addition. In all solvents tested solvent - $Al[OC(CF_3)_3]_3$ and $HOC(CF_3)_3$ were observed leaching off of the surface, except in the cased of cyclohexane.

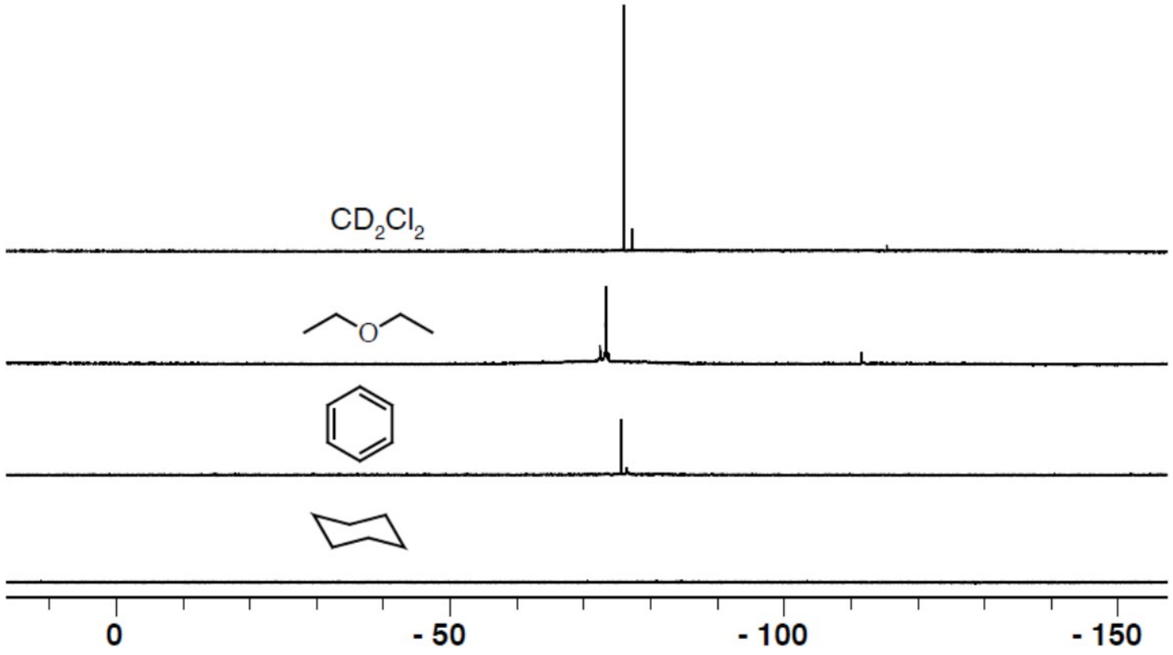

Figure S18. $^{19}F\{^{1}H\}$ NMR of **1** suspended in the indicated solvents.

Leaching experiment: 1 (200 mg) was placed in a teflon – valved flask then acetonitrile (2 mL) was transferred to the flask under vacuum at -196 °C. The slurry was stirred at room temperature for 30 minutes. The solution was canulae transferred under argon flow into a clean teflon – valved flask. The remaining solid was dried under vacuum for 1 hour at room temperature. In a glovebox, hexafluorobenzene (10 µL, 86 µmol) was added to the acetonitrile solution then the solution was transferred to an NMR tube and examined by $^{19}F\{^1H\}$ solution NMR with perfluorobenzene (-164 ppm) as an internal standard. From this experiment **1** contains 0.045 ± 0.004 mmol/g of PhF (-114 ppm). Signals for $Al(OR^F)_3$ (-76 ppm) and HOR^F (-73 ppm) are also present in this spectrum. The presence of these signals in the ^{19}F NMR spectrum indicate that the bridging silanol sites in **1** are not stable in the presence of MeCN. Desorption of $Al(OR^F)_3$ occurs in the presence of MeCN, and an unknown decomposition to form HOR^F occurs under these conditions.

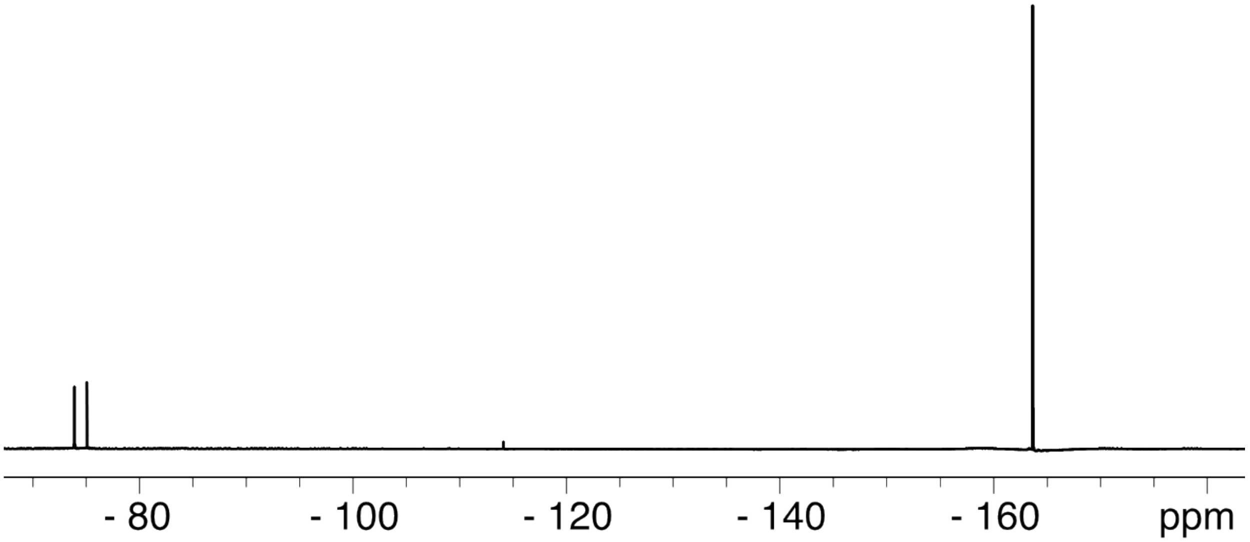

Figure S19. $^{19}F\{^1H\}$ NMR spectrum from the leaching experiment.

Hydrodefluorination of 1-adamantylfluoride with 3: **3** (20 mg, 4.8 μmol of Al) and 1-adamantylfluoride[16] (40 mg, 0.26 mmol) were loaded into a teflon-valved flask then it was connected to a vacuum line. The flask evacuated, cooled to 0 °C, and cyclohexane (2 mL) and triethylsilane (0.05 mL, 0.31 mmol) were added by syringe. The reaction was stirred at 0 °C for 2 hours. Under these conditions, 0.086 mmol Et_3SiF forms, giving a TON of 18.

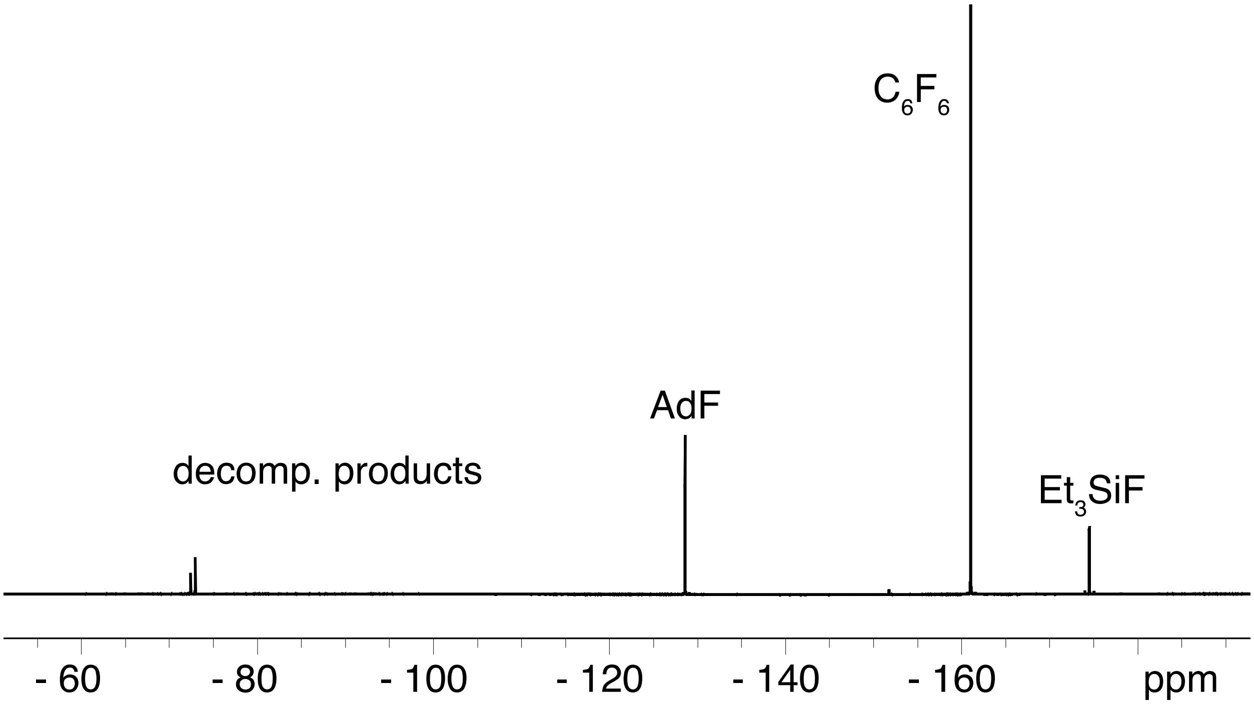

Figure S20. $^{19}F\{^1H\}$ NMR spectrum of the HDF of 1-adamantylfluoride with **3** after 2 hours.

DFT Calculations:

$SiO_{2\text{-}700}$ was modeled with a literature T8 silsesquixoane cluster adapted from Del Rosal et al, modified to include only one terminal hydroxyl group.[17] The geometries of all structures were optimized in Gaussian 09 with the B3LYP functional and 6-31G(d,p) basis set.[18] Frequency calculations at this level of theory produced no imaginary frequencies, indicating a ground-state energy minima equilibrium structure. The NMR parameters of **1** were calculated in using the GIAO method at the M06L/Al(6-311G(d,p)), 6-31G(d,p) level of theory. The ^{1}H and ^{27}Al chemical shifts are referenced to tetramethylsilane and $[Al(H_2O)_6]_3^+$ at the same level of theory. The molecular coordinates are provided at the end of this SI.

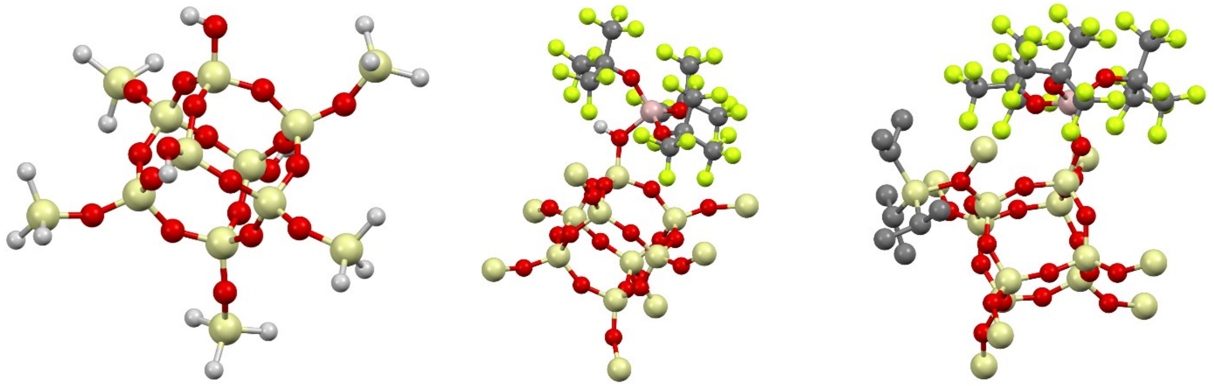

Figure S21. Calculated structures of the silica cluster model (left), **1-DFT** (middle), and **3-DFT** (right), with hydrogens from $-SiH_3$ groups and hydrocarbons on **1-DFT** and **3-DFT** hidden for clarity.

Gas-Phase Acidity:

Gas-Phase Acidity (GPA) calculations were performed with the calculated geometries in Gaussian 09 at the BP86/def2-TZVP level of theory. The geometries of HBr and H_2SO_4 were optimized at the B3LYP / 6-31G** level of theory. Bromine was described with the SDD basis set. The gas-phase acidity (GPA) is defined as:

$$HA \quad \text{---->} \quad H^+ \quad + \quad A^-$$

$$\Delta G\ (H^+ + A^-) - \Delta G\ (HA) = \text{GPA in kJ/mol}$$

The GPA produced at the B3LYP / 6-31G** level of theory did not reproduce experimental trends. With these geometries, other levels of theory were explored, particularly the BP86 functional, which has been known to produce accurate GPA values.[19] The experimental value as well as the theory that best reproduces the trend is used in the text and bolded in Table S2.

Table S2. Gas-Phase Acidity Screenings for HBr and H2SO4

	**HBr**	**H_2SO_4**
**Expt.**	**318**	**302**
B3LYP/6-31G**	310	325
B3LYP/6-31++G**	310	302
**BP86/def2-tzvp**	**321**	**306**
BP86/6-31++G**	311	302
BP86/def2-svp[&]	335	329
BP86-def2-tzvp[&]	335	316

[&] Geometries were optimized at the BP86 / def2-svp level of theory in Gaussian 09.

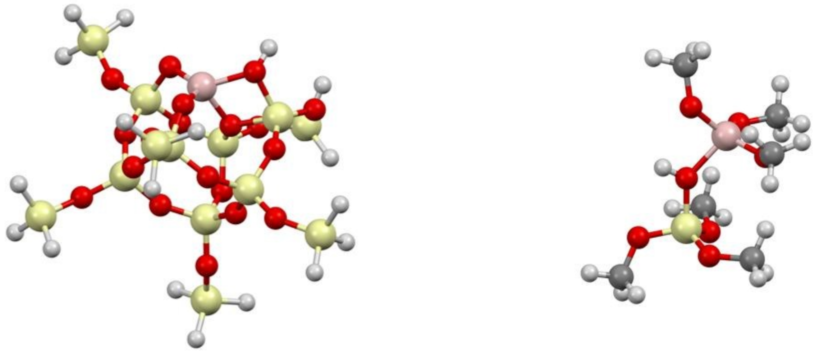

1-Al_{bridge}
279 kcal/mol

1-Al_{small}
299 kcal/mol

Figure S22. Simplified models for bridging silanols in SiO_2/Al_2O_3 and their calculated GPA.

NMR Calculations:

The NMR parameters of **1-DFT** and **3-DFT** were calculated using either B3LYP or M06-L functionals. For optimizations at M06-L / 6-31G**, the scaling factor of 0.952 for M06-L / 6-31+G** from Truhlar and coworkers is used.[20] Geometry optimizations of **1-DFT** and **3-DFT** using the 6-31G** basis set showed that both functionals give optimized structures with very similar ν_{OH} and Al–O*H* distances, indicating that these geometries of these species are similar at these levels of theory. However, the ^{27}Al C_Q was underestimated using this basis set. We screened basis sets and functional combinations and found that M06L with a 6-311G** basis set on Al, 6-31G** on other atoms, gave results that most closely agreed with experiment. These data are summarized in Table S3. All combinations of basis set and functional give ^{1}H NMR chemical shifts for the bridging silanol close to experiment.

Table S3. DFT Calculated Parameters for **1-DFT**.

**Opt. Theory**	<---------------------------- **B3LYP / 6-31G**** ---------------------------->					<-------------- **M06-L / 6-31G**** -------------->		
**NMR Theory**	**B3LYP / 6-31G****	**B3LYP / Al#**	**B3LYP / 6-311G****	**M06-L / Al#**	**M06-L / 6-311G****	**M06-L/ 6-31G****	**M06-L/ Al#**	**M06-L/ 6-311G****
**^{27}Al C_Q**	13.6 MHz	16.2 MHz	15.9 MHz	15.3 MHz	15.1 MHz	13.9 MHz	16.5 MHz	16.4 MHz
**δ(^{1}H) of Al(O*H*)**	4.85 ppm	4.79 ppm	4.99 ppm	5.12 ppm	5.18 ppm	4.83 ppm	4.87 ppm	4.64 ppm
**Al-O*H*(Å)**	<------------------------------ 2.46 Å ------------------------------>					<-------------- 2.47 Å -------------->		
**ν_{OH}**	<---------------------------- 3550 cm^{-1} ---------------------------->					<-------------- 3551 cm^{-1} -------------->		

Al is described with 6-311G**, while all other atoms are described with the 6-31G**.

Similarly, the δ(^{29}Si) was not well reproduced with B3LYP. Combinations of basis sets and functionals to calculate the ^{29}Si NMR chemical shift of **3-DFT** are given in Table S4. Similar to the data shown in Table 3, we found that M06L with a 6-311G** basis set on Al, 6-31G** on other atoms, gave results that most closely agreed with experiment.

Table S4. DFT Calculated Parameters for **3-DFT**.

**Opt. Theory**	<--------- **B3LYP / 6-31G**** --------->			<--- **M06-L / 6-31G**** --->		
**NMR Theory**	**B3LYP / 6-31G****	**B3LYP / 6-311G****	**M06-L / Al#**	**M06-L/ 6-31G****	**M06-L/ Al#**	**M06-L/ 6-311G****
**δ(^{29}Si)**	79 ppm	106 ppm	67 ppm	61ppm	61 ppm	25 ppm

Al is described with 6-311G**, while all other atoms are described with the 6-31G**.

Coordinates:

$SiO_{2\text{-}700}$ Cluster

O	3.42028200	-1.99133300	1.37257800
Si	2.11180900	-1.26103800	0.76348400
O	2.58651300	-0.13003500	-0.31802100
Si	2.20454600	1.14766300	-1.26823000
O	3.55158600	1.79447700	-1.88635800
O	1.15849900	-2.36762600	0.02236000
Si	-0.16943600	-2.59656200	-0.90689600
O	-0.26005600	-4.15996000	-1.30629800
O	1.28291000	-0.54253400	1.97539200
Si	-0.04160400	0.27993700	2.47598200
O	-0.04343800	1.76555500	1.79298200
Si	0.07095900	2.67791800	0.44355000
O	-1.23210400	2.41370600	-0.50914900
Si	-2.22127900	1.34451000	-1.25104200
O	-1.42258400	0.62414100	-2.48327900
Si	-0.08509500	-0.20256700	-2.94627300
O	1.23540000	0.65385000	-2.48889100
O	-0.05764900	-1.67997400	-2.25758400
O	-1.50283000	-2.16391000	-0.06705600
Si	-2.32617800	-1.04523100	0.79488600
O	-2.71162700	0.21436700	-0.17265100
O	-0.09439800	-0.44502900	-4.56052100
O	-0.02548100	0.41615600	4.08660500
O	-1.39452900	-0.52647600	2.03861400
O	1.43105200	2.28252100	-0.37982600
O	-3.69123200	-1.69282200	1.36979600

O	0.12572100	4.23536700	0.87220100
O	-3.50599400	2.12414000	-1.84923200
Si	0.87785700	0.59334300	5.46707100
Si	0.67452300	5.76595400	0.54431000
Si	-5.13533000	2.41313900	-1.71449100
H	-0.19783400	0.33590400	-5.11482200
Si	4.22018200	-3.42662900	1.61084100
Si	-0.51972500	-5.27338800	-2.51102600
H	0.61002800	-5.26250000	-3.47284800
H	-0.61497500	-6.60739400	-1.87230400
H	-1.78226500	-4.97577800	-3.23242900
H	4.54623300	-4.06068700	0.30890800
H	5.47284300	-3.12349000	2.34241100
H	3.38829800	-4.35776000	2.41331000
H	-5.47212300	2.86833900	-0.34214900
H	-5.48332200	3.47441200	-2.68874400
H	-5.90534900	1.18282800	-2.02400800
H	-0.14681500	6.71982400	1.32640100
H	2.09825700	5.89339500	0.94333500
H	0.54469200	6.06877300	-0.90363000
H	-0.02295100	1.07546100	6.54037000
H	1.46495000	-0.71160400	5.86093900
H	1.97069800	1.57588500	5.25480400
Si	-4.45633400	-2.42512300	2.64798800
H	-3.62343100	-3.52682600	3.19312400
H	-4.72151000	-1.43562600	3.72149100
H	-5.73986100	-2.97317400	2.14976700
Si	5.20390000	1.72839900	-2.02197900
H	5.62752800	0.42138700	-2.58403400

H	5.62317300	2.81947300	-2.93314900
H	5.84025800	1.91793800	-0.69446900

1-DFT

O	3.28552600	-1.23128800	3.85922100
Si	3.18167300	-0.74945400	2.32235000
O	2.41874400	-1.88720300	1.42792900
Si	1.85791600	-2.52003800	0.03893900
O	1.17374800	-3.94566000	0.30420900
O	2.35448500	0.65839900	2.22914500
Si	1.59759400	1.80362100	1.34733800
O	0.79803000	2.82939000	2.28289600
O	4.69156900	-0.52318900	1.73225400
Si	5.51602500	-0.04104300	0.39988200
O	5.20059600	-1.06459700	-0.83578500
Si	4.17751000	-1.79365800	-1.87579400
O	3.38493900	-0.65727000	-2.74737600
Si	2.59101600	0.75666100	-2.89984500
O	1.05965100	0.56282400	-2.29864000
Si	0.35261200	0.05503100	-0.92705400
O	0.75249400	-1.47639000	-0.60216800
O	0.54702300	1.05536200	0.31745900
O	2.69015900	2.61317300	0.43960400
Si	3.92645100	2.49193400	-0.62410400
O	3.35350700	1.92005000	-2.04829500
O	-1.31984400	0.09330000	-1.25553000
O	7.09982600	-0.03195400	0.71378300
O	5.05227800	1.46679600	-0.02862600
O	3.08161300	-2.68230400	-1.03827600
O	4.57575200	3.94574600	-0.88555800
O	5.00578200	-2.75216200	-2.87551400

O	2.46278000	1.16722200	-4.45218500
Si	8.35731300	-0.57681700	1.65133900
Si	5.31594300	-4.27237600	-3.46542800
Si	2.81362900	2.20472800	-5.70285900
H	-1.59063800	-0.15880900	-2.15810100
Si	2.48446400	-1.85036400	5.17973700
Si	0.34682800	3.53779600	3.71500800
H	-1.06613400	3.22484900	4.00890300
H	1.20896500	3.02839200	4.81167000
H	0.52678300	5.00330000	3.58575000
H	2.16989800	-3.28220400	4.95587800
H	3.38828200	-1.71621500	6.34651500
H	1.23169600	-1.09659900	5.42522200
H	4.28283200	2.31647400	-5.87577300
H	2.20466500	1.64338300	-6.93088700
H	2.24419600	3.54603600	-5.42635300
H	6.34477600	-4.14319500	-4.52339400
H	5.82087600	-5.15114200	-2.38130800
H	4.08094000	-4.86295200	-4.03904100
H	9.61889500	-0.23796800	0.95197200
H	8.32886300	0.08720900	2.97778800
H	8.26842200	-2.04713600	1.83563800
Si	5.66682100	5.11765600	-0.44615600
H	5.71074300	5.25841500	1.03101000
H	7.01755900	4.76055400	-0.94528800
H	5.23074000	6.39461300	-1.05757400
Si	0.34875900	-5.36200900	0.06629400
H	0.02602300	-5.94734100	1.38732900
H	-0.89327300	-5.10691800	-0.69928200

H	1.21068800	-6.30252300	-0.69304700
Al	-2.77196700	0.11745400	-0.01719100
O	-4.01261200	-0.64006700	-0.95685300
O	-3.01857300	1.77085200	0.40914000
O	-2.01370500	-0.81722700	1.24393700
C	-4.35168200	-1.56949100	-1.88728900
C	-3.56207000	-2.90530200	-1.67038500
C	-4.04287000	-0.99628200	-3.30937300
C	-5.88715700	-1.84350500	-1.75691700
C	-3.39936900	2.99967200	-0.04217700
C	-4.94299700	3.01779300	-0.30792300
C	-2.62805000	3.35720500	-1.35338900
C	-3.05213300	4.04002900	1.07554600
C	-2.34509100	-1.22907900	2.50364300
C	-1.83027600	-0.18561600	3.54965000
C	-1.66188700	-2.61063100	2.75313500
C	-3.89704600	-1.38131300	2.64336900
F	-3.97737900	-3.53099400	-0.56278300
F	-3.68633800	-3.74590100	-2.70838000
F	-2.24676200	-2.63624900	-1.51226800
F	-2.67703600	-0.92132900	-3.48874400
F	-4.52321900	-1.74795200	-4.30082600
F	-4.50661000	0.24506200	-3.43777300
F	-6.20384300	-2.09172300	-0.48208000
F	-6.58947300	-0.77448700	-2.16146400
F	-6.26100200	-2.89888200	-2.50400600
F	-5.61406200	2.90090000	0.84882700
F	-5.29292000	1.98280800	-1.08818500
F	-5.34067000	4.14841800	-0.91515200

F	-3.02547200	2.54401000	-2.35885000
F	-2.82607300	4.62455700	-1.73876800
F	-1.30663900	3.16771600	-1.19719400
F	-3.47771900	3.60772600	2.26681700
F	-3.61371700	5.23671500	0.83025000
F	-1.71916400	4.22086400	1.15687100
F	-0.55536100	0.15423500	3.28318700
F	-2.57105600	0.93295500	3.49127900
F	-1.87471100	-0.65996700	4.80554500
F	-1.88398000	-3.43443300	1.71802000
F	-0.32715100	-2.46671100	2.87770400
F	-2.12392200	-3.19768400	3.86909400
F	-4.33469100	-2.49141100	2.03325000
F	-4.49146600	-0.32788000	2.03161100
F	-4.30515400	-1.40833400	3.91683000

Deprotonated 1-DFT

O	3.41725100	-0.75615400	3.89489000
Si	3.26649800	-0.40063900	2.31709500
O	2.45629200	-1.58940800	1.55520600
Si	1.91426900	-2.42032300	0.25657600
O	1.32863700	-3.84119800	0.75621200
O	2.50163900	1.03213500	2.15132100
Si	1.74233400	1.96205500	1.02564400
O	0.83885200	3.07322100	1.76769100
O	4.77895400	-0.28220700	1.68437500
Si	5.62280400	0.02436100	0.31594900
O	5.37665200	-1.16401800	-0.77874400
Si	4.28017300	-1.96519700	-1.69442600
O	3.52766800	-0.92166600	-2.69513400
Si	2.63974100	0.42045400	-3.00357800
O	1.11190100	0.23990400	-2.47115800
Si	0.31287700	-0.06697600	-1.05245900
O	0.80231300	-1.56409400	-0.55459500
O	0.88110600	1.01701500	0.04304300
O	2.89366200	2.74434200	0.14199400
Si	4.03032900	2.42255900	-0.98202700
O	3.37216800	1.69633300	-2.28358900
O	-1.26208500	0.01704900	-1.25327300
O	7.20751200	0.08433700	0.68239300
O	5.19038200	1.46078900	-0.32745700
O	3.19777400	-2.72452600	-0.73498700
O	4.72221000	3.81391300	-1.45958300
O	5.08225500	-3.07378400	-2.57469100

O	2.62189900	0.67040800	-4.61103100
Si	8.29885600	-0.32825000	1.85611300
Si	5.03937200	-4.64592500	-3.08711300
Si	1.56263300	0.85468800	-5.87397200
Si	2.54956500	-1.20686300	5.23656900
Si	0.43413300	3.97510200	3.09132700
H	-0.94512500	3.68128100	3.52968100
H	1.37143500	3.68927900	4.21175300
H	0.54644200	5.41523900	2.74398700
H	2.17478000	-2.64040500	5.15799700
H	3.43302100	-1.00915300	6.41513600
H	1.33121900	-0.37858500	5.39455200
H	2.37431300	1.08265000	-7.09737500
H	0.73987400	-0.36605500	-6.06582200
H	0.67026100	2.02171500	-5.66369900
H	6.08388800	-4.81140900	-4.12911400
H	5.32954400	-5.57735900	-1.96646500
H	3.71681100	-4.99110700	-3.66872500
H	9.65942100	-0.10781700	1.30441200
H	8.12756700	0.51816600	3.06463200
H	8.16148000	-1.75563600	2.24521800
Si	6.03723900	4.80016300	-1.29788300
H	6.37146300	5.02754000	0.13253500
H	7.22409000	4.21619600	-1.97543100
H	5.71663600	6.10329600	-1.93155700
Si	0.39703700	-5.19850200	0.63163300
H	0.03733500	-5.65874600	1.99511700
H	-0.82556500	-4.95187100	-0.16329300
H	1.19138000	-6.27332800	-0.02522100

Al	-2.61292100	0.11229000	-0.13999500
O	-4.01972000	-0.77469700	-0.71133500
O	-3.07035900	1.78047600	0.19637200
O	-1.99604800	-0.56944700	1.37250900
C	-4.40943400	-1.78455100	-1.52173500
C	-3.66704900	-3.11732000	-1.16901700
C	-4.14121900	-1.43224300	-3.02624700
C	-5.94678100	-1.98264000	-1.30925800
C	-3.45925300	2.94525800	-0.37143600
C	-5.00186200	2.91866600	-0.65119500
C	-2.69124000	3.21822100	-1.70777200
C	-3.14562300	4.09573300	0.64543400
C	-2.30002000	-0.84866700	2.65813900
C	-1.75327100	0.28976100	3.58655700
C	-1.60018700	-2.19849500	3.03123300
C	-3.84360100	-0.99421500	2.88340200
F	-4.11884100	-3.63295800	-0.01178300
F	-3.82373300	-4.05879900	-2.12364600
F	-2.35103400	-2.89306700	-1.02376000
F	-2.84086600	-1.58263700	-3.33223400
F	-4.85018600	-2.21805400	-3.86770500
F	-4.47581300	-0.15938700	-3.27598300
F	-6.24542300	-2.01096600	-0.00438000
F	-6.63615900	-0.96958000	-1.86564500
F	-6.39417100	-3.13355000	-1.85908600
F	-5.68655700	2.91646300	0.50799400
F	-5.34444900	1.81629900	-1.32846300
F	-5.41684600	3.98641900	-1.36794000
F	-3.08920100	2.35462700	-2.66040300

F	-2.90131400	4.46861400	-2.17239000
F	-1.36968700	3.06280500	-1.54289600
F	-3.55207800	3.76692200	1.87703900
F	-3.74452800	5.25768800	0.30696500
F	-1.81858100	4.33209900	0.70419100
F	-0.49101600	0.61252600	3.24454800
F	-2.50092400	1.39849300	3.47054000
F	-1.73734000	-0.06625900	4.88919000
F	-1.85397600	-3.13111700	2.10055900
F	-0.25950600	-2.04216500	3.08764600
F	-2.00316600	-2.68116000	4.22356800
F	-4.30246800	-2.13408100	2.33993900
F	-4.49436900	0.02621100	2.30658400
F	-4.16914600	-1.00476400	4.19391300

1-Al$_{bridge}$

O	4.28974600	-0.48212700	0.18931200
Si	2.71212400	-0.32225300	-0.10670000
O	2.40434500	1.23091600	-0.50571900
Si	1.20864200	2.19428600	-1.10882600
O	1.87016700	3.50616300	-1.78595500
O	2.28903300	-1.32614100	-1.35659500
Si	0.99216600	-2.19633300	-1.83652500
O	0.89117600	-3.71423100	-1.99798400
O	1.84260300	-0.73388400	1.21940000
Si	0.48199700	-0.65469200	2.11882200
O	-0.28112000	0.76760300	1.89001800
Si	-0.93636400	2.01302800	1.05488900
O	-2.14826300	1.44885900	0.10713100
Si	-2.98458500	0.27750400	-0.68056200
O	-2.36255200	0.10816000	-2.19435100
O	0.33360300	1.33867600	-2.17866200
O	0.36291000	-1.26569300	-3.27725400
O	-0.29824900	-1.35682500	-0.95294900
Si	-1.35043700	-2.04986000	0.22963100
O	-2.70144300	-1.14435500	0.13404500
O	0.85257300	-0.86422000	3.67401300
O	-0.54332100	-1.86351700	1.63584800
O	0.22894200	2.68651200	0.12047100
O	-1.63962300	-3.58236700	-0.10822000
O	-1.52105700	3.11165600	2.08495200
O	-4.55979400	0.60852300	-0.67914700
Si	0.48131800	-1.49246600	5.16548500
Si	-1.46217200	4.66414100	2.66936300

Si	-6.06547100	0.60144600	-1.37743400
Si	5.66744900	-1.40801500	0.16964500
H	6.20524700	-1.48693400	-1.21203000
H	6.66289700	-0.75376800	1.05298200
H	5.38171400	-2.77770800	0.66724600
H	-7.06239300	0.86605500	-0.31282400
H	-6.14437900	1.66279300	-2.41241200
H	-6.33822000	-0.71763300	-2.00319200
H	-2.45765400	4.76854400	3.76363500
H	-0.10825900	4.96899500	3.19900900
H	-1.79853300	5.63492400	1.59686300
H	-0.95439700	-1.27415800	5.47758200
H	0.77425400	-2.94757600	5.18937600
H	1.32142800	-0.79738800	6.17020000
Si	-1.19763200	-5.19106400	-0.30093400
H	0.14655300	-5.44965800	0.25676700
H	-2.19884900	-5.96016400	0.48731300
H	-1.31985600	-5.57342800	-1.72222900
Si	3.23155700	4.33274800	-2.24396700
H	4.07331100	3.49729700	-3.13955800
H	2.79817800	5.55089700	-2.97098300
H	4.02805200	4.72394700	-1.05273700
Al	-0.64939500	-0.04749900	-2.28810000
H	0.05537500	-1.86581600	-3.97695200

1-Al$_{small}$

Si	1.75057600	0.01172100	-0.11926700
O	0.18546900	0.02315300	-0.79873400
O	2.66539100	0.11808900	-1.46733600
O	2.08815800	-1.37521200	0.65522000
O	1.80742400	1.24130800	0.94130700
C	1.59303600	-1.72640800	1.95874500
H	1.93605300	-2.74111000	2.17188700
H	1.99304100	-1.04374200	2.71450500
H	0.49950800	-1.69246300	1.97545100
C	4.05320100	-0.21752500	-1.54467200
H	4.25930200	-1.16253600	-1.03312500
H	4.31258500	-0.31635200	-2.60106200
H	4.66772500	0.57439100	-1.10289000
C	1.32596000	2.57842200	0.72813600
H	1.52926900	3.14274700	1.64078100
H	1.85665200	3.05367800	-0.10426400
H	0.24995200	2.56757300	0.53140200
Al	-1.64645100	-0.00252300	-0.14827300
O	-1.89156900	1.59873500	0.45256100
O	-1.70826600	-1.23051200	1.07180200
O	-2.18646600	-0.39816200	-1.75177400
C	-2.53954700	1.99640100	1.63953500
H	-1.91428400	2.71137700	2.19317500
H	-3.49152900	2.49824700	1.41450700
H	-2.75097100	1.15190300	2.31006200
C	-2.11176000	-2.57244000	0.91661600
H	-1.27265800	-3.25753800	1.10883300
H	-2.90744300	-2.81832800	1.63356600

H	-2.49056000	-2.78838800	-0.09261800
C	-3.36632700	-0.03164300	-2.42549800
H	-4.16513000	-0.77268100	-2.27326400
H	-3.74971900	0.94731800	-2.10205600
H	-3.17890600	0.02863300	-3.50615000
H	0.10441000	-0.19405300	-1.74298600

3-DFT

O	3.29663600	0.12006100	3.82748200
Si	3.08404900	-0.29706500	2.28287400
O	2.16085100	-1.62376400	2.16382000
Si	1.29659500	-2.67740500	1.24028700
O	0.35653100	-3.57728700	2.17109900
O	2.36319600	0.98950700	1.50949900
Si	1.96508900	1.49348200	0.02689100
O	1.38151700	3.13033500	0.10276900
O	4.52771000	-0.53555400	1.55668700
Si	5.37167300	-1.18600600	0.30914300
O	4.78230800	-2.66101800	-0.06217500
Si	3.53879000	-3.53919700	-0.67512700
O	2.96395100	-2.79260800	-2.00721600
Si	2.30765400	-1.62344800	-2.93614300
O	0.81409100	-1.23342800	-2.40140900
Si	0.03424400	-0.77182800	-1.02682700
O	0.45725600	-1.80984300	0.15889200
O	0.72029200	0.71405100	-0.61619100
O	3.24920600	1.58601400	-0.96046400
Si	4.22052000	0.66968500	-1.93828800
O	3.28823900	-0.30218100	-2.85070000
O	-1.52418800	-0.59390500	-1.23589600
O	6.93152100	-1.28578400	0.71355100
O	5.22537200	-0.19527000	-0.98971900
O	2.35404200	-3.65412800	0.44674000
O	5.05939500	1.70104800	-2.85993200
O	4.09092100	-5.01061900	-1.04946200

O	2.23565000	-2.10277800	-4.47745400
Si	8.22625300	-2.25374300	1.09888300
Si	3.79449000	-6.64488500	-1.09481400
Si	1.22557000	-2.57746200	-5.71343400
Si	2.68456800	0.00064800	5.37540300
Si	-0.23405100	3.35178900	-0.73530300
H	-0.32255700	4.81539200	-0.69715700
H	-0.02127900	2.85861900	-2.09948200
H	-1.20573300	2.65650300	0.09473600
H	2.10788900	-1.34460200	5.60239800
H	3.81280300	0.23217800	6.30842100
H	1.64920800	1.04097900	5.57960400
H	2.08784000	-3.00730700	-6.83988000
H	0.37037200	-3.70825000	-5.27826100
H	0.37369900	-1.44214600	-6.14317800
H	4.84811400	-7.26658900	-1.93116300
H	3.84921600	-7.21063600	0.27574400
H	2.46103400	-6.91536000	-1.68713800
H	8.60797300	-3.08710900	-0.06745000
H	9.35233900	-1.36399200	1.46731000
H	7.88913100	-3.13387800	2.24519200
Si	5.93327000	1.85673200	-4.27036300
H	6.89463600	2.96766300	-4.07538500
H	6.66529800	0.60235400	-4.57143600
H	5.01674700	2.18273000	-5.38957600
Si	-0.31522600	-4.61799200	3.26602400
H	-0.21331700	-4.03725800	4.62710600
H	-1.73128000	-4.86053300	2.92134400

H	0.43193900	-5.90164400	3.23267800
Al	-2.88073000	-0.08814200	-0.23258300
O	-4.17247700	-1.25671200	-0.15515200
O	-3.44221200	1.48187700	-0.81161200
O	-2.13578600	0.21955500	1.35187800
C	-4.51003500	-2.55743900	-0.33981000
C	-3.66771900	-3.48638100	0.59507600
C	-4.28425800	-2.98831500	-1.82891100
C	-6.02749600	-2.70269300	0.01487400
C	-4.14414800	2.14102200	-1.76317700
C	-5.68027600	1.86076700	-1.62331400
C	-3.66342300	1.74336000	-3.19950900
C	-3.89481200	3.67182000	-1.54152400
C	-2.41647300	0.50648700	2.64655000
C	-1.81746900	1.91277300	2.98667100
C	-1.73749600	-0.56787400	3.56154900
C	-3.95833400	0.52189500	2.93453600
F	-4.04754100	-3.35304800	1.87718500
F	-3.77640400	-4.78603200	0.26353400
F	-2.36434400	-3.15123100	0.52567900
F	-2.97275500	-3.16816300	-2.08051500
F	-4.91825300	-4.13856500	-2.12705800
F	-4.73068100	-2.03904500	-2.66166200
F	-6.30112000	-2.08602700	1.17085100
F	-6.78978000	-2.14342100	-0.94111600
F	-6.39387500	-3.99480700	0.13462400
F	-6.14103800	2.39890100	-0.47971300
F	-5.92220500	0.54752200	-1.58909400

F	-6.38279900	2.38948900	-2.64514400
F	-4.06923300	0.50239400	-3.50766200
F	-4.12844500	2.58393400	-4.14349200
F	-2.31857900	1.75609900	-3.26605600
F	-3.99218100	3.99373600	-0.24945700
F	-4.72540000	4.45402200	-2.24148500
F	-2.62207500	4.00750700	-1.93070200
F	-0.53868600	1.99922000	2.55016800
F	-2.51033400	2.88739500	2.36705800
F	-1.80541300	2.18112800	4.30252600
F	-2.03956000	-1.80271900	3.14145600
F	-0.38829700	-0.44804000	3.51722000
F	-2.11058700	-0.45526500	4.84822400
F	-4.45011300	-0.72484300	2.97123200
F	-4.59553600	1.18926600	1.95965200
F	-4.24815500	1.11865100	4.10654500
Si	2.40879900	4.61718600	0.54382500
C	1.26684900	5.87674200	1.42613500
H	1.98930800	6.27855500	2.15504800
C	3.77340600	4.18041600	1.79937700
H	4.17808600	5.20448200	1.90347600
C	3.21445100	5.11795600	-1.10747800
H	3.79490800	4.22761300	-1.38759800
C	4.95037100	3.30596100	1.32597600
H	4.67916100	2.25163100	1.26751100
H	5.33392000	3.60548700	0.34647900
H	5.77657500	3.38645000	2.04149100
C	3.29149700	3.76460400	3.20418500

H	2.81554900	2.78262900	3.19215200
H	4.14826600	3.70666800	3.88526800
H	2.58535000	4.48142100	3.63212700
C	0.11543500	5.26492100	2.25284700
H	-0.72313300	4.95953700	1.62176700
H	0.41944700	4.39113300	2.83278100
H	-0.27446600	6.01217000	2.95297700
C	0.77322100	7.08698200	0.60848000
H	0.01313000	6.80763900	-0.12643700
H	0.31407300	7.82181100	1.27976500
H	1.58286500	7.59406100	0.07611500
C	2.26980200	5.42836000	-2.28475400
H	1.64189000	4.57478000	-2.55417000
H	1.61525700	6.28057300	-2.07996100
H	2.85870300	5.67813200	-3.17494500
C	4.21753300	6.27749200	-0.90324300
H	4.98045900	6.04973600	-0.15326300
H	4.73981500	6.48580900	-1.84372500
H	3.71881200	7.20539900	-0.60284800

References:

1. Copéret, C.; Comas-Vives, A.; Conley, M. P.; Estes, D. P.; Fedorov, A.; Mougel, V.; Nagae, H.; Núñez-Zarur, F. and Zhizhko, P. A. Surface Organometallic and Coordination Chemistry toward Single-Site Heterogeneous Catalysts: Strategies, Methods, Structures, and Activities. *Chemical Reviews* **2016**, 116, 323–421.
2. Le Roux, E.; Chabanas, M.; Baudouin, A.; De Mallmann, A.; Copéret, C.; Quadrelli, E. A.; Thivolle-Cazat, J.; Basset, J.-M.; Lukens, W.; Lesage, A.; Emsley, L. and Sunley, G. J. Detailed Structural Investigation of the Grafting of [Ta(=CH*t*Bu)(CH_2*t*Bu)$_3$] and [Cp*$TaMe_4$] on Silica Partially Dehydroxylated at 700 °C and the Activity of the Grafted Complexes toward Alkane Metathesis. *J. Am. Chem. Soc.* **2004**, 126, 13391-13399.
3. Müller, L. O.; Himmel, D.; Stauffer, J.; Steinfeld, G.; Slattery, J.; Santiso-Quiñones, G.; Brecht, V. and Krossing, I. Simple Access to the Non-Oxidizing Lewis Superacid PhF→Al(ORF)$_3$ (R^F = C(CF_3)$_3$). *Angew. Chem.* **2008**, 120, 7772 –7776; *Angew. Chem. Int. Ed*. **2008**, 47, 7659 –7663.
4. Cory, D. G. and Ritchey, W. M. Suppression of Signals from the Probe in Bloch Decay Spectra. *Journal of Magnetic Resonance* **1988**, 80, 128-132.
5. Gan, Z. H. Measuring multiple carbon-nitrogen distances in natural abundant solids using R-RESPDOR NMR. *Chem Commun* **2006**, 45, 4712-4714.
6. Chen, L.; Wang, Q. A.; Hu, B. W.; Lafon, O.; Trebosc, J.; Deng, F. and Amoureux, J. P. Measurement of hetero-nuclear distances using a symmetry-based pulse sequence in solid-state NMR. *Phys Chem Chem Phys* **2010**, 12, 9395-9405.
7. Brinkmann, A. and Kentgens, A. P. M. Proton-Selective 17O-1H Distance Measurements in Fast Magic Angle Spinning Solid-State NMR Spectroscopy for the Determination of Hydrogen Bond Lengths. *J. Am. Chem. Soc*. **2006**, 128, 14758-14759.
8. Bak, M.; Rasmussen, J. T. and Nielsen, N. C. SIMPSON: A General Simulation Program for Solid-State NMR Spectroscopy. *J. Magn. Reson*. **2000**, 147, 296-330.
9. Tosner, Z.; Andersen, R.; Stevenss, B.; Eden, M.; Nielsen, N. C. and Vosegaard, T. Computer-Intensive Simulation of Solid-State NMR Experiments using SIMPSON. *J. Magn. Reson.* **2014**, 246, 79-93.
10. Tosner, Z.; Vosegaard, T.; Kehlet, C.; Khaneja, N.; Glaser, S. J. and Nielsen, N. C. Optimal Control in NMR Spectroscopy: Numerical Implementation in SIMPSON. *J. Magn. Reson.* **2009**, 197, 120-134.
11. Trebosc, J.; Hu, B.; Amoureux, J. P. and Gan, Z. Through-space R^3-HETCOR experiments between spin-1/2 and half-integer quadrupolar nuclei in solid-state NMR. *J. Magn. Reson.* **2007**, 186, 220-227.
12. Venkatesh, A. Hanrahan, M. P. and Rossini, A. J. Proton detection of MAS solid-state NMR spectra of half-integer quadrupolar nuclei. *Solid State Nuclear Magnetic Resonance* **2017**, 84, 171-181.
13. Yao, Z.; Kwak, H. T.; Sakellariou, D.; Emsley, L. and Grandinetti, P. J. Sensitivity enhancement of the central transition NMR signal of quadrupolar nuclei under magic-angle spinning. *Chem. Phys. Lett.* **2000**, 327, 85-90.
14. van Meerten, S. G. J.; Franssen, W. M. J. and Kentgens, A. P. M. ssNake: A cross-platform open-source NMR data processing and fitting application. *Journal of Magnetic Resonance* **2019**, 301, 56-66.

15. Feike, M.; Demco, D. E.; Graf, R.; Gottwald, J.; Hafner, S.; and Spiess, H. W. Broadband multiple-quantum NMR spectroscopy. *J Magn Reson Ser A* **1996**, 122, 214-221.
16. Olah, G. A., and Watkins, M. Fluorinations with Pyridinium Polyhydrogen Fluoride Reagent: 1-Fluoroadamantane. *Org. Synth.* **1978**, 58, 75.
17. Del Rosal, I.; Gerber, I. C.; Poteau, R.; Maron, L. J. Phys. Chem. A 2010, 114, 6322-6330.
18. Gaussian 09, Revision A.02, M. J. Frisch, G. W. Trucks, H. B. Schlegel, G. E. Scuseria, M. A. Robb, J. R. Cheeseman, G. Scalmani, V. Barone, G. A. Petersson, H. Nakatsuji, X. Li, M. Caricato, A. Marenich, J. Bloino, B. G. Janesko, R. Gomperts, B. Mennucci, H. P. Hratchian, J. V. Ortiz, A. F. Izmaylov, J. L. Sonnenberg, D. Williams-Young, F. Ding, F. Lipparini, F. Egidi, J. Goings, B. Peng, A. Petrone, T. Henderson, D. Ranasinghe, V. G. Zakrzewski, J. Gao, N. Rega, G. Zheng, W. Liang, M. Hada, M. Ehara, K. Toyota, R. Fukuda, J. Hasegawa, M. Ishida, T. Nakajima, Y. Honda, O. Kitao, H. Nakai, T. Vreven, K. Throssell, J. A. Montgomery, Jr., J. E. Peralta, F. Ogliaro, M. Bearpark, J. J. Heyd, E. Brothers, K. N. Kudin, V. N. Staroverov, T. Keith, R. Kobayashi, J. Normand, K. Raghavachari, A. Rendell, J. C. Burant, S. S. Iyengar, J. Tomasi, M. Cossi, J. M. Millam, M. Klene, C. Adamo, R. Cammi, J. W. Ochterski, R. L. Martin, K. Morokuma, O. Farkas, J. B. Foresman, and D. J. Fox, Gaussian, Inc., Wallingford CT, 2016.
19. Bohrer, H., Trapp, N., Himmel, D., Schleep, M., Krossing, I. *Dalton Trans.*, **2015**, *44*, 7849-7499.
20. Zhang, W., Ma, Y., Santos-Lopez, A., Lownsbury, J. M., Yu, H., Liu, W-G., Truhlar, D. G., Campbell, C. T., Vilches, O. E. *J. Am. Chem. Soc.* **2018**, *140*, 328-338.
